# Expert-Consistency-Guided Proposal Refinement for 3D Object Detection from Sparse Radar Point Clouds

**DOI:** 10.3390/s26196339

**Published:** 2026-10-08

**Authors:** Zixuan Liu, Wei Xiong

**Affiliations:** 1 Detroit Green Technology Institute, Hubei University of Technology, Wuhan 430068, China; 2411621306@hbut.edu.cn; 2 School of Electrical and Electronic Engineering, Hubei University of Technology, Wuhan 430068, China; 3 Department of Computer Science and Engineering, University of South Carolina, Columbia, SC 29201, USA

**Keywords:** 3D object detection, radar point cloud, autonomous driving, proposal refinement, expert consistency, geometric voting

## Abstract

Sparse radar point clouds provide limited evidence for three-dimensional (3D) object detection, making weak proposals especially sensitive to ranking and suppression. Standard post-processing depends on the primary detector’s scores and geometry and can therefore remove useful candidates. We introduce an expert-consistency-guided refinement framework that acts on detector outputs without changing the backbone. Candidate boxes are retained and reweighted according to agreement with an independently trained radar expert; supported boxes are then adjusted by local geometric voting. A separate high-performance route instead trains a localization-quality-aware confidence head with relaxed positive assignment and does not use the expert-consistency gate. The framework is evaluated on Astyx, MAN TruckScenes, V2X-Radar-V, and K-Radar with three training seeds. At a 3D intersection-over-union threshold of 0.50, the strict route improves average precision (AP) in all 12 matched dataset–seed comparisons, with a mean gain of 0.9726 AP. The high-performance route, whose gain comes from this quality-aware ranking branch rather than from expert-consistency gating, raises macro AP from 35.3552 to 39.1214, a gain of 3.7662 AP. These improvements are observations under the evaluated protocol: the strict voting parameters were selected using the same dataset–seed cells, and leave-one-dataset-out threshold selection yields 11/12 positive held-out differences. The strict route also shows metric-dependent confidence–calibration trade-offs and requires an additional expert detector and voting computation. The results therefore support protocol-specific gains, not a guarantee of improved reliability on unseen data.

## 1. Introduction

Automotive perception must remain reliable when poor illumination, adverse weather, or difficult viewing geometry limits camera observations. Millimeter-wave radar is valuable in these conditions because it directly measures range and radial velocity. It also remains operational in situations that can degrade passive optical sensing. Recent four-dimensional (4D) radar datasets cover a broader range of driving, weather, and cooperative-perception settings [1,2]. These developments have made radar-only three-dimensional (3D) object detection useful beyond its conventional role in camera–radar fusion. A radar-only detector can provide an independent source of spatial and motion evidence when visual information is unavailable or unreliable.

The same measurements that make radar useful also produce a difficult input for 3D detection. Radar point clouds contain far less geometric evidence than dense light detection and ranging (LiDAR) scans, and an object may generate only a few irregular returns. Their number, amplitude, and Doppler distribution vary with distance, aspect, occlusion, sensor configuration, and pre-processing [3,4]. Reflections may cover only part of the visible object surface and need not coincide with its geometric center. Return density also falls with range and can change markedly between measurement systems. A detector must therefore reconstruct a complete 3D box from partial, noisy, and measurement-dependent observations.

Existing 3D detectors handle irregular point sets through point-based, voxel-based, or pillar-based representations [5,6,7,8,9]. PointPillars is particularly suitable for controlled radar experiments because it converts occupied pillars into an efficient bird’s-eye-view representation. Radar-specific work has also explored denser representations and multimodal fusion to compensate for missing geometry [10,11,12]. These approaches improve the evidence available to the detector, but they do not remove the uncertainty of the final proposal set. Radar-only detection still has to decide which weak hypotheses are useful without relying on image semantics or dense LiDAR geometry.

This decision becomes important after the network has generated its candidate boxes. Several uncertain proposals may correspond to the same object, yet score filtering and non-maximum suppression (NMS) can remove them before their combined evidence is considered. Hard NMS favors a single high-scoring box, even when neighboring boxes contain complementary localization information. Binary objectness scores create a related problem because they do not distinguish positive boxes by localization quality. Geometric averaging is not a general solution either: nearby predictions should contribute only when their agreement is credible. Soft suppression and quality-aware scoring address parts of this process [13,14,15]. They do not jointly determine which sparse-radar proposals should remain available, how they should be ranked, and when their geometry can be refined safely.

We separate this problem into proposal availability, proposal quality, and proposal consistency: Availability determines whether weak evidence survives long enough to be examined. Quality concerns whether confidence reflects the localization of a positive box rather than object presence alone. Consistency describes whether neighboring predictions and an independent detector support the same object hypothesis. These properties are connected but not interchangeable. Retaining more boxes does not improve their ordering, and averaging retained boxes can be harmful when local support is unreliable. A proposal-level method must therefore preserve evidence without treating every neighbor as equally trustworthy.

To study these decisions directly, we developed an expert-consistency-guided proposal-refinement framework that leaves the detector backbone unchanged. A candidate-preserving stream retains primary and complementary evidence that would otherwise be removed during early filtering. An independently trained radar expert then reweights the retained proposals according to cross-model agreement. Local voting uses these reweighted scores to adjust primary proposals with overlapping neighbors, while leaving residual proposals unchanged. A separate localization-quality-aware branch aligns confidence with 3D intersection over union and provides an accuracy-oriented operating point. Both routes use the same PointPillars-style primary backbone. The strict route nevertheless adds an expert network, so its total model capacity and inference cost are greater than those of the primary detector alone.

Unlike overlap-only Soft-NMS, the expert gate uses another detector’s confidence and geometry to assess primary hypotheses. Unlike direct box fusion, it does not add expert boxes to the output or average their coordinates into primary boxes. This preserves the identity of weak primary hypotheses while changing their ranking, which is relevant when sparse radar returns leave several plausible localizations. These distinctions describe the operation, not a guarantee of superiority over ensembling; the comparison axes are discussed in the method description.

Average precision (AP) alone does not show whether an improvement is repeated across datasets and training initializations. This distinction matters for radar because the evaluated datasets differ in return density, sensing geometry, coverage, and pre-processing. We therefore report two predefined endpoints: the strict route emphasizes positive paired differences across all dataset–seed comparisons, whereas the high-performance route emphasizes macro average precision. This separation makes the trade-off between consistent improvement and higher average accuracy explicit.

The main contributions of this study are as follows:

1. We formulate sparse-radar detection as a problem of proposal availability, localization-aware ranking, and geometric consistency. The resulting framework operates without changing the detector backbone.
2. We use cross-model consistency to reweight retained proposals and use the reweighted scores in restricted local voting. A separate quality-aware route provides an operating point for higher average accuracy.
3. We evaluate both routes on four radar dataset conversions using three training seeds. Under the selected evaluation protocol, the strict route improves all 12 matched comparisons, while the high-performance route raises macro AP from 35.3552 to 39.1214.

The remainder of this paper reviews related work, describes the two refinement routes, and reports the experimental protocol and results. The Section 7 discuss the observed mechanisms, practical boundaries, and computational cost.

## 2. Related Work

### 2.1. Radar-Based 3D Detection and Representations

Automotive radar data can be encoded as spectra, range–angle maps, dense tensors, or post-detection point clouds. Each representation retains a different part of the measurement signal. Dense formats keep responses that may disappear during point extraction, at the cost of larger grids and specialized encoders. Post-detection point clouds are compact and compatible with established 3D detectors, but they remain sparse and dependent on the sensor and pre-processing pipeline. Networks trained on radar spectra and dense tensors have shown that these measurements can support learned detection [10,16]. We use point-cloud conversions to provide the same proposal interface across all evaluated datasets. This also fixes the stage at which the methods are compared. Raw-signal encoding remains part of each dataset conversion, while refinement begins only after the detector has decoded its boxes.

Point-based, voxel-based, and pillar-based detectors handle irregular point sets in different ways. PointNet and PointNet++ operate directly on unordered points [5,6]. VoxelNet, SECOND, and sub-manifold sparse convolutions regularize the input while avoiding computation over most empty cells [7,8,17]. PointPillars encodes vertical pillars and scatters them into a bird’s-eye-view (BEV) feature map [9]. Two-stage and center-based detectors add further geometric reasoning to improve proposal features [18,19,20]. Regardless of the backbone, final AP still depends on the ordering and removal of predicted boxes. We therefore retain a fixed PointPillars-style backbone and study the proposal decisions made after prediction.

### 2.2. Recent Radar-Only Detectors and Fusion Scope

RadarPillars studies radar-specific velocity encoding, pillar attention, and network scaling for sparse point-cloud detection [21]. RadarNeXt combines a re-parameterizable point-cloud detector with multi-path deformable feature enhancement [22]. These methods primarily improve feature extraction, whereas our controlled study concerns decoded proposal preservation, ranking, and restricted geometric updates. This difference in intervention point is not evidence that proposal refinement outperforms their radar-specific architectures. Protocol-matched numerical comparisons are needed to establish that relationship.

RADE-Net instead consumes projections of range–azimuth–Doppler–elevation tensors [23]. A compact post-detection point cloud does not retain those spectral measurements; directly substituting our five-channel point inputs would change the method. Consequently, published results from tensor-input and point-input systems are not interchangeable under the present conversion and evaluator.

Parallel and attention-based fusion architectures motivate combining complementary information from several computational paths. However, the relationship here is conceptual. Our primary detector and expert observe the same radar modality; the expert contributes decoded predictions rather than camera features or another sensor stream. The method is therefore radar-only prediction-level evidence combination, not multimodal sensor fusion such as camera–radar feature integration [11]. For example, the MHA-PCNN architecture retains visible and near-infrared feature paths and combines parallel CNN branches with hybrid attention [24]. That classification application does not provide detector evidence for radar; it is relevant only to the general design idea that alternative paths may be retained before evidence combination. Multiple paths may share the same errors, and neither attention nor separate training alone guarantees complementary information. The expert gate must consequently be distinguished both from single-stream post-processing and from general two-detector ensembling.

### 2.3. Candidate Preservation and Expert-Guided Refinement

Two-stage detectors treat proposals as an explicit intermediate representation. Faster R-CNN generates regions before classification and box regression, while Cascade R-CNN refines localization over successive stages [25,26]. VoteNet applies learned Hough voting to aggregate point evidence around object centers [27]. These architectures differ from a single-stage radar detector, but they share the idea that several uncertain observations can support a more stable object hypothesis.

Non-maximum suppression (NMS) turns a dense collection of hypotheses into a ranked detection list. Hard NMS removes overlapping boxes immediately. Soft-NMS instead reduces their scores [13], and learned NMS models duplicate removal as a proposal-reasoning problem [28]. Sparse R-CNN learns a fixed proposal set and updates its elements iteratively [29]. Suppression is especially consequential for sparse radar because different boxes may be supported by different subsets of the few returns from one object. Keeping only the highest-scoring box can discard useful localization evidence. Keeping every box creates duplicates and admits unsupported hypotheses. A bounded proposal set offers a practical compromise and leaves candidate evidence available for a later reliability check. These methods also intervene at different stages. Soft-NMS changes inference scores, learned NMS requires another trained module, and Sparse R-CNN changes the detector architecture. Our experiments instead apply refinement rules to a fixed detector output.

Box voting estimates consensus geometry from neighboring predictions rather than selecting a single box. Weighted box fusion uses confidence-weighted coordinates for a related purpose [30]. Three-dimensional boxes require additional care because centers, dimensions, and periodic headings cannot be averaged in the same way. In our framework, preservation keeps candidate evidence available, while voting is restricted to proposals with independent expert support. The expert supplies only a support signal and does not add boxes to the final output. Training the expert independently also avoids deriving both a proposal score and its supporting evidence from the same prediction stream. M1 and M2 retain candidate evidence, M3 changes localization-aware ranking, and expert-guided voting is applied only after local agreement has been established.

### 2.4. Confidence and Localization Quality

Localization quality can be measured by intersection-over-union (IoU).

Target assignment controls which locations learn objectness and box regression. FCOS and ATSS use explicit or adaptive assignment rules [31,32]. Focal Loss addresses class imbalance, while IoU-Net, Generalized Focal Loss, VarifocalNet, and TOOD incorporate localization quality or task alignment into the ranking score [14,15,33,34,35]. The distinction matters in sparse radar: a box may contain strong evidence of an object while its center, heading, or dimensions remain inaccurate. Since NMS and thresholding follow the confidence order, this mismatch can affect AP. Small changes in a sparse-radar box can also produce large changes in 3D IoU. The quality target must therefore be computed from decoded positive boxes rather than assigned as a fixed class label. We study localization-aware scoring as a separate training branch, while retaining an objectness signal for assigned positives.

Quality-aware ranking and confidence calibration are related but distinct. Average precision depends mainly on ordering, while calibration compares the reported confidence with empirical correctness [36]. Detection calibration can additionally depend on location, scale, class, and localization uncertainty [37,38]. We report expected calibration error as a diagnostic for the accuracy-oriented route. The strict route is assessed through paired AP because its purpose is to test whether each matched evaluation improves, not whether confidence values are calibrated.

### 2.5. Radar Benchmarks and Multimodal Context

K-Radar provides 4D radar measurements in varied weather, and Enhanced K-Radar examines density reduction and data accessibility [1,39]. View-of-Delft and TJ4DRadSet add urban road users and driving scenes [40,41]. V2X-Radar includes vehicle, infrastructure, and cooperative viewpoints [2]. These datasets differ in coverage, return density, sensing geometry, and labeling. Results obtained from one dataset or conversion may therefore reflect a particular measurement regime. We use the same detector family and matched training seeds across four conversions. The comparisons are kept paired within each dataset rather than combined into a shared leaderboard. Dataset-level AP values remain in their original evaluation contexts, while paired changes show whether an effect has the same direction across measurement regimes.

Multimodal detectors combine radar measurements with image features, spatial fusion, temporal alignment, or cross-modal objectives [11,12,42,43,44,45,46,47]. Image features can supply semantic information when radar observations are weak. The radar-only setting used here removes that source of information and makes proposal credibility depend on radar evidence alone.

Prior work has established strong backbones, adaptive assignment, localization-aware scores, suppression methods, and public radar benchmarks. Their interaction at the proposal level has received less attention under matched multi-seed and multi-dataset evaluation. We study that interaction with a fixed PointPillars-style backbone, the candidate-preserving baseline, denoted RDAR, as the reference, and separate routes for average accuracy and paired consistency.

## 3. Proposed Method

### 3.1. Problem Formulation

#### Method Roles and Provenance

The method contains three functionally distinct components. M1 and M2 are proposal-availability operations applied after a standard PointPillars-style detector has produced boxes: M1 preserves plausible candidates, and M2 recovers a conservatively scored residual tail. M3 is a training-time localization-quality-aware confidence branch. The strict route additionally uses a separately trained radar expert and restricted local voting; these are proposal-level refinement operations, not changes to the detector backbone. PointPillars, NMS, and the box parameterization are adopted detector or post-processing primitives, whereas candidate preservation, expert gating, and the constrained voting rule are the proposal-refinement contributions studied here. Detailed implementation values are provided for reproducibility; the conceptual roles and their evaluation order are the primary method claim.

Let a radar frame be represented by a point set $X={\left\{x_{n}\right\}}_{n=1}^{N_{x}}$, where each point contains spatial coordinates and the radar attributes supplied by the corresponding dataset conversion. The detector maps *X* to a set

(1) $$P={\left\{\left(b_{i},s_{i},c_{i}\right)\right\}}_{i=1}^{N_{p}},$$

where $b_{i}=\left(x_{i},y_{i},z_{i},l_{i},w_{i},h_{i},\theta_{i}\right)\in R^{7}$ is a 3D oriented box, $s_{i}\in \left[0,1\right]$ is its confidence, and $c_{i}$ is its class. The experiments consider the car class and use the dataset-specific training and validation partitions fixed for each dataset conversion.

Conventional training minimizes classification, regression, and direction losses, whereas inference additionally applies score filtering and NMS. Our objective is not simply to increase the number of raw candidates. We seek a ranked set that satisfies three properties: plausible weak candidates remain available for recovery, confidence preferentially ranks better-localized boxes, and redundant hypotheses can provide geometric consensus rather than only suppressing one another. Reliability is then assessed by paired AP differences across datasets and seeds.

We distinguish proposal availability, proposal quality, and proposal consistency. Availability concerns whether a candidate reaches the final processing stages. Quality concerns whether its score agrees with its localization. Consistency concerns whether neighboring predictions support a common geometry. M1 and M2 primarily address availability, M3 addresses quality, and strict consistency refinement acts on the final proposal set. The division is functional: M1, M2, and the final gate/vote are proposal-level operations, while M3 changes training.

### 3.2. Baseline Detector and Framework Overview

The common detector follows PointPillars [9]. Points are grouped into vertical pillars with a voxel size of [0.25,0.25,5.0] m within the baseline range [0,−25,−3,60,25,2] m. At most 32 points are retained per pillar, with 16,000 training voxels and 40,000 test voxels. A PillarVFE maps each occupied pillar to 64 channels, PointPillarScatter places the features on the BEV grid, and a 2D backbone with block depths [3,5,5], strides [2,2,2], and channels [64,128,256] produces the detection features. Three upsampling branches return 128 channels each and are concatenated before the anchor head.

The baseline head predicts classification, seven box residuals, and a two-bin direction label. Its loss weights are 1.0 for classification, 2.0 for localization, and 0.2 for direction. For the fixed Astyx configuration, the car anchor is approximately [4.003,1.800,1.510] m with bottom height −0.180 m, and the positive/negative matching thresholds are 0.60/0.45. Dataset-specific configuration files provide the corresponding data paths and anchor statistics, while the network family and evaluation rule remain fixed.

The method first builds a shared proposal stream and then evaluates two endpoints. M1 retains weak but plausible primary proposals, and M2 adds a conservatively scored residual tail. Together, these two stages define the candidate-preserving RDAR baseline used throughout the experiments.

The strict route starts from the RDAR primary proposals. It uses an independently trained radar expert only as a support signal, applies an expert-consistency gate, and then performs restricted local voting on supported primary proposals; the box dimensions remain unchanged. The high-performance route is separate: it trains the M3 quality-aligned confidence head and uses relaxed positive assignment with the accuracy-oriented prior. Voting is not appended to this configuration.

**Figure 1:**
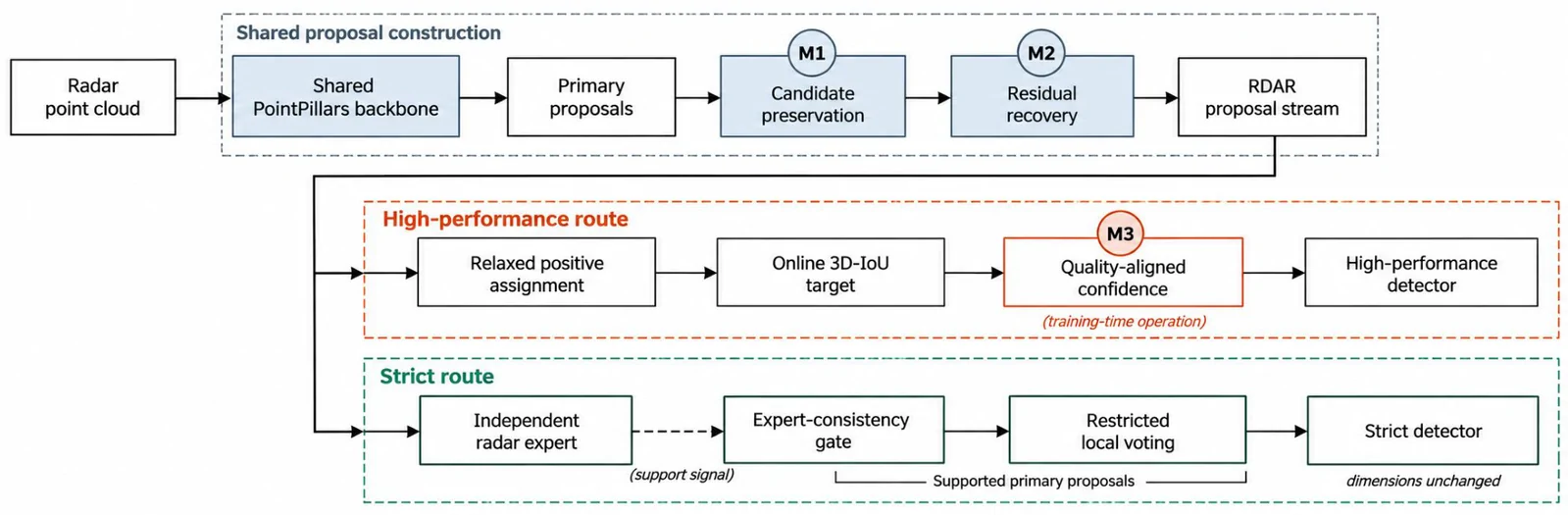
Overview of the proposal-refinement framework. A shared PointPillars backbone produces the primary proposal stream. M1 preserves candidate availability, and M2 recovers a conservatively scored residual tail, forming the candidate-preserving baseline, denoted RDAR. The high-performance route uses relaxed positive assignment and an online three-dimensional intersection-over-union (3D IoU) target to train the M3 quality-aligned confidence head. The strict route uses an independently trained radar expert as a support signal, gates primary proposals by expert consistency, and applies restricted local voting only to supported primary proposals; box dimensions are unchanged.

The shared proposal construction and the two operating routes are summarized in Figure 1.

The seed-2028 construction analysis follows the construction of the high-performance route, and the three-seed construction analysis repeats the M1–M3 chain across three seeds. By contrast, the formal strict comparison starts from RDAR-compatible proposals and selects for positive differences in all 12 dataset–seed pairs. The two analyses therefore measure different operating objectives.

### 3.3. Candidate Preservation and Recovery

#### 3.3.1. M1: Candidate-Preserving NMS

The baseline configuration uses a score threshold of 0.10 and a very strict NMS IoU threshold of 0.01. Such a setting produces a compact output, but it can delete useful evidence before any complementary proposal is considered. M1 lowers the score threshold to zero and increases the NMS threshold to 0.50. The pre-NMS and post-NMS limits remain 4096 and 500, respectively. Thus, M1 changes which boxes remain available, not the maximum reported proposal count. The design intent is to delay irreversible deletion until complementary evidence has been considered. M1 changes candidate availability rather than detector capacity or the final proposal budget.

Let π order boxes by score. Hard NMS accepts the highest remaining box and removes a box *j* whenever

(2) $${\mathrm{IoU}}_{3D}\left(b_{\pi (i)},b_{j}\right)>\tau_{\mathrm{nms}}.$$

Increasing $\tau_{\mathrm{nms}}$ from 0.01 to 0.50 delays this deletion and retains moderately overlapping alternatives. This resembles the preservation motivation of Soft-NMS [13], although our implementation does not use continuous score decay. The retained alternatives become inputs to recovery, quality-aware ranking, and voting.

Removing the initial score threshold does not mean that every proposal is promoted to a high-confidence detection. The post-NMS limit remains finite, subsequent ranking uses the learned score, and residual additions in M2 are explicitly capped below the positive primary-score range. M1 is therefore a candidate-access operation: it creates the opportunity for later modules to use weak boxes without treating them as correct detections.

#### 3.3.2. M2: Residual Proposal Recovery

M1 cannot restore a hypothesis that exists only in a complementary prediction stream. M2 takes a primary set $P=\{\left(b_{i},s_{i}\right)\}$ and a residual set $R=\{\left(r_{j},t_{j}\right)\}$. Frame identifiers and ordering are validated before fusion. The residual boxes are sorted by $t_{j}$, and only the top $K_{r}=50$ are processed.

The design intent is conservative recovery. A residual may contribute geometry when it supports a primary proposal, but its score must remain below the primary lane so that complementary evidence cannot override a confident primary prediction.

For each residual proposal, we find

(3) $$i^{⋆}\left(j\right)=\arg\max_{i}{\mathrm{IoU}}_{3D}\left(r_{j},b_{i}\right).$$

When the best IoU is at least $\tau_{r}=0.10$, the residual and primary geometries are combined using normalized score weights. For the six non-angular box parameters,

(4) $${\tilde{b}}_{j,1:6}=\frac{s_{i^{⋆}}b_{i^{⋆},1:6}+t_{j}r_{j,1:6}}{s_{i^{⋆}}+t_{j}}.$$

Heading is π-periodic for an oriented rectangular box. We therefore use doubled-angle circular averaging:

(5) $${\tilde{\theta }}_{j}=\frac{1}{2}\mathrm{atan}2\left(\sum_{k\in \{i^{⋆},j\}}w_{k}\sin2\theta_{k},\sum_{k\in \{i^{⋆},j\}}w_{k}\cos2\theta_{k}\right).$$

This avoids cancellation when equivalent headings fall on opposite sides of the numerical angle boundary. If the best overlap is below 0.10, the residual geometry is appended unchanged.

Recovered proposals are deliberately prevented from dominating the primary ranking. Let $s_{\min}^{+}$ be the smallest positive primary score in the evaluated prediction set and $t_{\max}$ the maximum residual score in the frame. The assigned recovery score is

(6) $$s_{j}^{\mathrm{rec}}=\left(\frac{s_{\min}^{+}}{2}\right)\left(0.5+0.5\frac{t_{j}}{\max(t_{\max},\epsilon )}\right),$$

where adjacency denotes multiplication and $\epsilon =10^{-12}$ prevents division by zero. The factor in parentheses lies in [0.5,1], so every residual score is at most half the smallest positive primary score.

### 3.4. Localization-Quality-Aware Confidence (M3 Branch)

M3 is an accuracy-oriented comparison branch, not a component of the primary expert-consistency endpoint. It is included because confidence ordering is a separate design axis from expert agreement; reporting it separately prevents the larger macro-AP gain from being mistaken for the main reliability claim.

Binary positive labels do not distinguish anchors by localization accuracy. M3 replaces the positive classification target with an online 3D-IoU target. For each positive anchor, the predicted residual and assigned regression target are decoded into $b_{i}$ and $g_{i}$. Their aligned quality is

(7) $$q_{i}={\mathrm{IoU}}_{3D}\left(b_{i},g_{i}\right)\in \left[0,1\right].$$

The IoU is detached from the classification graph, so the classification loss does not back-propagate through the IoU operation into box regression.

Directly using $q_{i}$ can make a valid positive behave nearly like a negative while the regressor is immature. We retain a residual objectness component:

(8) $${\hat{q}}_{i}=\rho +\left(1-\rho \right)q_{i}^{\gamma },\;\rho =0.55,\;\gamma =1.0.$$

Negatives receive zero and ignored anchors are excluded. The parameter ρ controls the minimum target for an assigned positive. At ρ=1, the target reduces to binary objectness; at ρ=0, it is pure IoU quality. The selected value retains both signals.

Let $\ell_{i}$ be the classification logit and $p_{i}=\sigma \left(\ell_{i}\right)$. The quality focal term is

(9) $$L_{\mathrm{QF}}=\frac{1}{N_{+}}\sum_{i\in C}{\left|{\hat{q}}_{i}-p_{i}\right|}^{\beta }\mathrm{BCEWithLogits}\left(\ell_{i},{\hat{q}}_{i}\right),\;\beta =2,$$

where the juxtaposition after the modulation factor denotes multiplication, C is the set of cared anchors, and $N_{+}$ is the number of positives, clipped below by one. This construction follows the general quality-aware principle of Generalized Focal Loss [14] but uses online aligned 3D IoU and an explicit objectness floor for sparse radar.

The full training loss is

(10) $$L=L_{\mathrm{QF}}+2L_{\mathrm{box}}+0.2L_{\mathrm{dir}}.$$

M3 does not add a second inference branch. The original classification logit is trained to express the combined objectness–quality target, leaving the 4.830 M parameter count unchanged. The M3-only control retains the baseline assignment configuration and uses the candidate-preserving post-processing settings. The separate high-performance route combines M3 with 0.50/0.35 positive/negative assignment thresholds and the accuracy-oriented prior configuration.

The design intent is to make the ranking score reflect localization quality without discarding objectness. The role of M3 is ranking: a well-localized proposal should receive a larger target than a poorly localized proposal assigned to the same class. Better ranking can improve which proposal survives NMS and where a detection appears on the precision–recall curve. It does not guarantee that confidence is calibrated in every sense, so expected calibration error (ECE) is measured independently in Section 5.4.

### 3.5. Expert-Guided Refinement

The strict route applies two proposal-level operations to a primary RDAR set: an expert-consistency quality gate, and local box voting. The expert is a stable BEV-gated detector with the same output parameterization. Its purpose is not to replace the primary detector but to provide a second assessment of whether a proposal is spatially supported. The strict route is selected by the paired no-regression gate described below, not by the performance of a single seed.

For reproducibility, the expert uses the same PointPillars-style radar input, class definition, box coder, and seven-parameter output head as the primary stream. Its configuration has a 64-channel PillarVFE, a 32-channel DensityAwarePillarGate in the pillar backbone, and a 64-channel BEV scatter. The BEV backbone uses three blocks with depths [3,5,5], strides [2,2,2], filters [64,128,256], and three 128-channel upsampling paths. After concatenation, the expert applies a stable squeeze–excitation gate with reduction 8 and an initial bounded residual scale of 0.1, followed by the multi-scale BEV context module with hidden width 32, dilations [1,2,4], and initial residual scale 0.1. The gate is channel-wise: for BEV feature *x*, it computes x↦x(1+r(2σ(g(GAP(x)))−1)), where *r* is a learned bounded scalar initialized to 0.1. At the pillar level, the density-aware gate normalizes each pillar feature $f_{i}$, forms a scene context $c_{i}^{\mathrm{pg}}$ by averaging normalized pillars from the same sample, and encodes the normalized point count $d_{i}=\mathrm{clip}\left(\log\left(1+n_{i}\right)/\log\left(33\right),0,1\right)$. Specifically, $f_{i}=\mathrm{LayerNorm}\left(f_{i}^{\mathrm{raw}}\right)$, and $c_{i}^{\mathrm{pg}}$ is computed only from pillars whose first coordinate in the voxel-coordinate tensor equals the batch index of *i*; different batch samples therefore never share context. If a sample has no occupied pillars, the module returns it unchanged. It then computes

(11) $$a_{i}=2\;\sigma \;\left(W_{2}\;\mathrm{GELU}\;\left(W_{1}\left[f_{i};c_{i}^{\mathrm{pg}};d_{i}\right]\right)\right)-1,\;f_{i}^{\prime }=f_{i}⊙\left(1+r_{p}a_{i}\right),$$

where $W_{1}$ and $W_{2}$ are biased linear layers with the framework’s default parameter initialization, the hidden width is 32, and $r_{p}$ is a learned scalar initialized to zero. The multi-scale BEV context first reduces the concatenated BEV tensor to 32 channels, applies depthwise 3×3 branches with dilations 1,2, and 4, concatenates the branch outputs, projects them back to the original channel count using a bias-free 1×1 convolution followed by batch normalization, and adds the residual $r_{c}h\left(x\right)$ with scalar $r_{c}=0.1$ at initialization. Each depthwise branch uses its own shared reduced input but does not share branch weights. These definitions make the expert modules explicit rather than relying only on YAML names. In the released implementation, these correspond to the classes DensityAwarePillarGate and MultiScaleBEVContext; the stable channel gate is StableSEGate2D. Their source files are density_aware_pillar_gate.py and base_bev_backbone.py in the released implementation. The expert is trained independently with the same fixed split, augmentation policy, optimizer, 160-epoch schedule, and checkpoint epoch as the paired primary configuration; no additional labels or sensor inputs are used. For the fixed-expert factorial analysis, the expert checkpoint is held fixed within each conversion: seed 2027 is used for Astyx, TruckScenes, and V2X-Radar-V, and seed 2028 is used for K-Radar, while the primary prediction stream varies over seeds 2026, 2027, and 2028. This prevents the expert from implicitly tracking the seed of each primary cell.

#### 3.5.1. Expert-Consistency Quality Gate

Let $E=\{\left(e_{j},u_{j}\right)\}$ be the expert predictions. The final $K_{r}=50$ entries of the combined primary set are reserved for residual additions and are not gated. The gate is a support test rather than a second detector decision: agreement raises a proposal’s rank, while disagreement lowers its rank without deleting the box. For every other primary proposal, the best expert match is

(12) $$j^{⋆}\left(i\right)=\arg\max_{j}{\mathrm{IoU}}_{3D}\left(b_{i},e_{j}\right).$$

If the maximum IoU $v_{i}$ is at least $\tau_{e}=0.30$, the score becomes

(13) $$s_{i}^{\prime }=s_{i}^{1-\alpha }u_{j^{⋆}}^{\alpha }v_{i}^{\eta },\;\alpha =0.30,\;\eta =0.25.$$

This geometric product requires agreement among primary confidence, expert confidence, and overlap. The small IoU exponent avoids allowing modest geometric noise to erase otherwise supported radar proposals. If $v_{i}<0.30$, the proposal is retained but downweighted:

(14) $$s_{i}^{\prime }=\kappa s_{i},\;\kappa =0.50.$$

Keeping unmatched proposals instead of deleting them preserves the conservative character of the pipeline. The gate affects ranking but does not move a box.

#### 3.5.2. Local Proposal Voting

The voting stage refines geometry using the gated scores. Its design intent is to correct small center and heading errors while avoiding changes to box size, which could create artificial IoU gains. For each primary box *i*, a neighborhood is defined by

(15) $$N_{i}=\left\{j:{\mathrm{IoU}}_{3D}\left(b_{i},b_{j}\right)\geq \tau_{v}\right\},\;\tau_{v}=0.24.$$

If $|N_{i}|\leq 1$, the box is unchanged. Otherwise, $w_{j}=\max\left(s_{j}^{\prime },10^{-8}\right)$, and

(16) $$c_{i,1:6}=\frac{\sum_{j\in N_{i}}w_{j}b_{j,1:6}}{\sum_{j\in N_{i}}w_{j}}$$

defines the consensus geometry. Consensus heading is again calculated in doubled-angle space. The strict xy mode updates only the BEV center:

(17) $$b_{i,xy}^{\prime }=\left(1-\lambda \right)b_{i,xy}+\lambda c_{i,xy},\;\lambda =0.40.$$

Heading is interpolated circularly with weights 1−λ and λ. Height, center *z*, length, width, and height remain unchanged. Restricting the update prevents the consensus from artificially shrinking or expanding boxes to satisfy the evaluation threshold.

The IoU neighborhood threshold is lower than the 0.50 evaluation threshold. This is intentional: multiple radar hypotheses for the same vehicle can have modest 3D overlap because of height and dimension noise while still agreeing in BEV. Threshold sensitivity is nevertheless evaluated over a closed grid rather than justified only after observing the selected setting. The final 50 residual proposals remain outside the voting pool, ensuring that consistency refinement cannot undo M2 by moving or suppressing the recovered tail.

The stages preserve a fixed division of responsibility. M1 determines which primary candidates survive, and M2 adds complementary residual proposals. In the strict route, expert consistency changes only the ranking of primary proposals. Local voting then updates only the BEV center and heading of supported primary boxes. Residual proposals never enter the gate or voting pool, and voting does not change box dimensions.

### 3.6. Operating Routes and Selection Criteria

The RDAR baseline denotes M1 followed by M2 under the fixed recovery protocol. The M3-only control applies M3 and provides the intermediate trainable comparison. The high-performance route uses the same quality-focal formulation with relaxed positive assignment and the accuracy-oriented prior configuration. It is chosen by macro AP across the four datasets. Its intended use is to show the performance available when average accuracy is prioritized.

The strict route applies the expert gate and local vote. Let $A_{d,r}^{m}$ be AP for method *m*, dataset *d*, and seed *r*. A candidate is strict only when

(18) $$\Delta_{d,r}=A_{d,r}^{m}-A_{d,r}^{\mathrm{RDAR}}>0\;\forall \left(d,r\right)\in D\times S,$$

where |D|=4 and |S|=3. This definition contains no tolerance for a small negative difference. It is stronger than requiring a positive macro mean, but it remains bounded to the tested data, splits, and seeds.

Selection by Equation (18) is not a claim that the strict route has the highest AP. Indeed, the high-performance route has a larger macro mean. The two routes answer different engineering questions: whether a configuration offers the largest average gain, and whether it avoids a regression in the available paired tests.

Table 1 lists the exact operations enabled by every reported configuration. It should be consulted when comparing the M3-only control, high-performance route, and strict route because they optimize different objectives and are not successive aliases for one model. In particular, relaxed positive assignment belongs to the high-performance route, whereas expert gating and local voting define the strict route.

### 3.7. Algorithmic Summary

For clarity, the complete procedure is summarized below.

Step 1: Encode the radar frame with PillarVFE, scatter pillar features to BEV, and predict anchor classification, box residuals, and direction.
Step 2: During M3 training, decode positive boxes, compute aligned 3D IoU, form the soft target in Equation (8), and optimize the combined loss. The baseline route retains binary classification.
Step 3: Apply candidate-preserving NMS with zero initial score threshold, $\tau_{\mathrm{nms}}=0.50$, and a 500-box post-NMS limit.
Step 4: Retain the top 50 complementary residual proposals. Fuse a residual box with its best primary match when 3D IoU is at least 0.10; otherwise, append it. Assign the conservative score in Equation (6).
Step 5: For the strict route, match each non-residual primary proposal to the expert stream, apply Equation (13), and downweight unmatched boxes by 0.50.
Step 6: Form local neighborhoods at $\tau_{v}=0.24$ and update BEV center and heading using Equation (17). Return the residual tail unchanged.
Step 7: Rank the resulting predictions and evaluate them with the identical ${\mathrm{AP}}_{R40}$ implementation used for every compared method.

### 3.8. Method Distinctions and Parameter Specification

The gate does not append expert boxes or replace primary coordinates with expert coordinates. It uses expert overlap and confidence to change the primary ranking, and then voting aggregates neighboring primary hypotheses. Thus, “supported” describes weighted cross-model/local agreement, not a guarantee that every updated box is a true positive or passes a separate binary expert-match mask. A box with no expert match remains eligible for local voting at its penalized score if it has a nontrivial neighborhood.

Table 2 compares the proposal operations and the evidence used for score and geometry refinement.

Table 3 summarizes the principal notation and its domains.

Table 4 summarizes the reported implementation settings and the displayed voting search grid.

The threshold-selection rule uses the evaluation differences themselves and, therefore, favors settings with positive observed gains. Its 12/12 count is a descriptive property of the selected configuration, not an unbiased estimate of future no-regression probability. Holding thresholds constant across seeds does not remove this selection dependence. The leave-one-dataset-out analysis addresses the final threshold choice separately, with the remaining design dependence stated alongside its results.

Earlier exploratory scripts additionally record a seed-2028 local sweep over $\tau_{v}\in \left\{0.23,0.24,0.25\right\}$ and λ∈{0.38,0.40,0.42,0.44,0.45}. The table therefore documents the displayed comparison grid and frozen settings, not a prospectively isolated hyperparameter search. Uncertainty summaries do not account for this broader development history.

### 3.9. Training and Implementation Details

Training uses the OpenPCDet codebase and an Adam one-cycle optimizer for 160 epochs. The fixed baseline configuration uses a batch size of four per graphics processing unit (GPU), peak one-cycle learning rate 0.003 (initial rate 0.0003 with division factor 10), weight decay 0.01, momentum 0.9, one-cycle momenta [0.95,0.85], warm-up fraction 0.4, division factor 10, and gradient norm clipping at 10. Random world flips, rotations in [−π/4,π/4], and scaling in [0.95,1.05] are applied during training. Ground-truth sampling is disabled in the fixed radar configuration used for the controlled comparisons.

All models are trained independently with seeds 2026, 2027, and 2028. The formal comparison uses the same data partitions, class definition, metric, checkpoint epoch, and evaluation code for each paired method. Proposal-level scripts validate frame order before combining prediction files. This check is important because an apparently valid fusion between misaligned frame lists could silently produce meaningless boxes.

The original progressive construction screen holds the seed at 2028 and adds M1, M2, quality alignment, and the high-performance calibration variant sequentially. The three-seed construction analysis uses the same result records to repeat the PointPillars–M1–M2–M3 chain for seeds 2026–2028. It reports all local regressions and is used to estimate stage variability, not to establish that every intermediate module is positive for every seed. The formal strict result instead evaluates the final strict route on all 12 paired cells.

Computational cost is reported in three parts: Trainable parameters distinguish model capacity. A fixed profiler measures detector forward execution, including OpenPCDet post-processing, but excluding AP computation and result serialization. A separate timer measures only the local voting loop and excludes prediction-file input/output. Keeping these measurements separate prevents a fast detector result from hiding an expensive post-processing stage.

### 3.10. Implementation Safeguards

The implementation uses four safeguards that define the scope of the claim. Prediction files must have identical frame order; residual scores are bounded by Equation (6) and kept in the final 50-entry tail, outside the gate and voting pool; and residual fusion and voting use doubled-angle averaging for the π-periodic heading. The strict vote updates only *x*, *y*, and heading, while *z*, *l*, *w*, and *h* remain unchanged. Finally, one global threshold setting is used across all four datasets; dataset-specific compensation screens are reported only as diagnostics. These constraints keep the comparison reproducible and prevent the reliability endpoint from becoming a collection of independently tuned dataset-specific procedures.

## 4. Experimental Protocol

To make the evaluation easy to follow, the protocol separates one primary endpoint from three supporting evidence layers. We first freeze the data conversions, splits, class mapping, and metric; we then define the RDAR reference and all comparison routes; next, we report the primary three-seed endpoints. Mechanism and control analyses explain where the gain comes from, robustness analyses test sensitivity and selection dependence, and practical analyses describe operating cost and failure boundaries. None of these diagnostic results is substituted for the primary endpoint.

### 4.1. Research Questions

We ask four questions: RQ1 tests whether the strict route improves RDAR in matched dataset–seed comparisons. RQ2 isolates the contribution of each component and compares the route with conventional post-processing controls. RQ3 examines robustness, calibration, threshold selection, and selection-aware uncertainty. RQ4 characterizes computational cost, proposal-budget trade-offs, and practical failure boundaries.

### 4.2. Datasets and Protocol

We evaluate radar-only car detection on four datasets. Astyx provides an automotive radar point-cloud benchmark [48]. MAN TruckScenes covers autonomous trucking scenes and a sensor geometry distinct from passenger-car platforms [49]. For V2X-Radar-V, we use the vehicle-side subset of a cooperative 4D radar collection. K-Radar contains 4D radar measurements acquired in varied weather [1,2]. The experiment records refer to the standardized dataset conversions as Astyx, TruckScenes-mini, V2X-Radar-V-400, and K-Radar-400. We use shorter names in the text. The experiment package retains the exact conversion names and fixed configuration settings specified above.

The fixed splits contain 300/100 train/validation frames for Astyx and 320/80 for each remaining conversion, with 531, 3950, 268, and 198 validation car instances, respectively. The records provide frame-level point-cloud, calibration, image, and annotation fields but no scene or sequence identifiers. We found no exact train–validation overlap in the available point-cloud IDs. We also applied a conservative adjacency check: a symmetric ±1 difference in point_cloud.pc_idx. It flagged 82/100 Astyx, 4/80 TruckScenes, 1/80 V2X-Radar-V, and 80/80 K-Radar validation frames. The converted records do not contain ordering metadata, so this check cannot establish temporal adjacency. It also cannot rule out correlations from the same scene or vehicle. Excluding all flagged frames would leave 18, 76, 79, and 0 validation frames, making K-Radar AP impossible to estimate. Our evaluation is therefore limited to the fixed frame-level protocol. We do not claim scene-disjoint, vehicle-disjoint, cross-initialization, or deployment generalization. The “mini” and “400” suffixes name the conversion protocol, not new dataset releases.

The primary metric is car ${\mathrm{AP}}_{R40}$ at 0.50 3D IoU, evaluated with a common implementation and class mapping. AP is reported on a 0–100 scale, so a difference of 1.0 is one AP point. Because AP is computed over the complete validation set and is not additive over frames, frame and instance counts describe evaluation scope rather than defining per-frame confidence intervals. The paired and cluster-resampling summaries describe only these finite fixed sets.

The formal experiment uses four datasets and three seeds (2026, 2027, and 2028). Each model is trained independently under the same schedule and evaluated with the same class mapping and code. We report the mean and sample standard deviation across seeds. For the strict route, comparisons are paired within each seed. The primary detector and expert are trained independently but use the same data protocol and seed. This pairing makes individual regressions visible without introducing a cross-seed difference. We examine that issue separately by holding the expert fixed across seeds. This diagnostic tests sensitivity, not initialization-independent robustness.

The main claims rely on the formal three-seed comparison. The original seed-2028 progressive screen and the three-seed construction analysis examine the construction path. Calibration, point dropout, threshold transfer, runtime, and qualitative cases probe model behavior and practical limits.

Table 5 summarizes the conversions, inputs, spatial ranges, and training seeds. Acquisition settings differ, but the class mapping, ${\mathrm{AP}}_{R40}$ metric, 0.50 3D-IoU threshold, and paired-seed rule are shared.

### 4.3. Baselines and Comparison Routes

The conventional PointPillars-style detector is the architectural reference. RDAR, comprising M1 and M2, is the paired baseline for the formal comparison; M3-only and the high-performance route isolate the training-time localization-quality contribution. The strict route adds expert-consistency score refinement and conditional geometry voting to RDAR-compatible primary proposals. We use two control families: fixed-output post-processing controls (standard box voting, Gaussian Soft-NMS, and weighted box fusion) and same-expert controls (pooled NMS, oriented WBF, score gating, and gate-plus-voting). The latter family keeps the expert prediction fixed while changing the combination rule, which makes the comparison more informative about the source of the gain.

### 4.4. Metrics and Statistical Summaries

For a ranked list at a fixed 3D-IoU match threshold, precision and recall after the first *k* predictions are

(19) $$\mathrm{Prec}\left(k\right)=\frac{\mathrm{TP}(k)}{\mathrm{TP}(k)+\mathrm{FP}(k)},\;\mathrm{Rec}\left(k\right)=\frac{\mathrm{TP}(k)}{\mathrm{TP}(k)+\mathrm{FN}(k)}.$$

Here, TP, FP, and FN denote true positives, false positives, and false negatives, respectively. ${\mathrm{AP}}_{R40}$ samples the interpolated precision envelope at 40 recall positions. Because confidence determines the traversal order, M3 can change AP even when the set of decoded boxes is similar. Because voting changes geometry, the strict route can also change which predictions cross the 0.50 IoU matching threshold.

The macro result for method *m* is the unweighted average of the four dataset-level seed means:

(20) $${\bar{A}}_{\mathrm{macro}}^{m}=\frac{1}{4}\sum_{d\in D}\left(\frac{1}{3}\sum_{r\in S}A_{d,r}^{m}\right).$$

This choice gives each dataset equal influence regardless of the number of frames or objects. It avoids allowing the largest validation partition to dominate, but it does not imply equal operational importance.

Standard deviations describe variation across the three seeds; they are not confidence intervals for dataset performance. For the strict comparison, we report all 12 paired differences, their mean and median, a bootstrap interval, and an exact sign test. We place greater weight on the effect sizes and the smallest paired gain. Threshold selection and evaluation use the same four-dataset protocol, so the result describes consistency within this protocol rather than held-out performance. Resampling the four dataset means gives a descriptive interval of [0.5464, 1.4834] AP. The nested cell holdout and max-statistic analyses below are therefore reported as selection-aware analyses, not independent external tests.

## 5. Results

### 5.1. Main Findings: Accuracy and Consistency

Table 6 compares the two operating endpoints with RDAR and the M3-only control over three seeds. The high-performance route is evaluated by macro AP and calibration, whereas the strict route is evaluated by its paired AP difference from RDAR in each dataset–seed cell.

We present the results in the same order as the research questions: primary accuracy and consistency first, mechanism and controls second, robustness and selection diagnostics third, and practical operating boundaries last. This ordering prevents the larger collection of diagnostic analyses from obscuring the primary endpoint.

**Figure 2:**
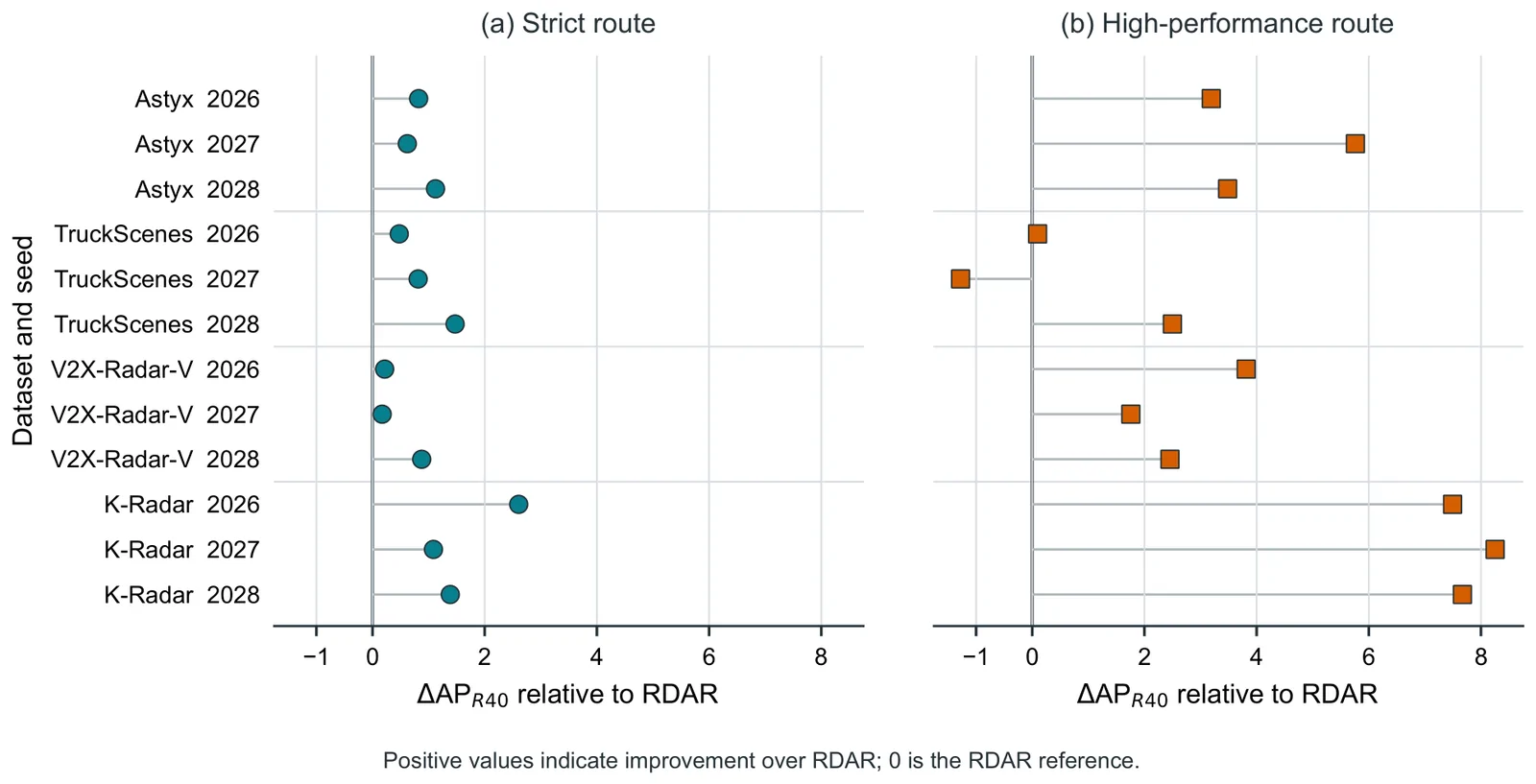
Paired AP differences relative to RDAR across the four datasets and three seeds. The strict route remains positive in every tested dataset–seed cell, whereas the high-performance route contains one negative cell. Strict route: 12/12 positive cells, mean +0.9726 AP, median +0.8493 AP, 95% bootstrap CI [+0.6468,+1.3536], range [+0.1718,+2.6060]. High-performance route: 11/12 positive cells, mean +3.7662 AP, one negative cell (TruckScenes, seed 2027). AP: average precision; CI: confidence interval; RDAR: the candidate-preserving baseline.

The strict route raises macro AP from 35.3552 to 36.3278 (+0.9726). It also improves all 12 matched dataset–seed cells. The high-performance route reaches 39.1214 macro AP (+3.7662), with its largest gain on K-Radar. Its only decrease is on TruckScenes at seed 2027, from 18.3845 to 17.1075 AP. The M3-only control reaches 38.7283 macro AP and has the same negative cell. The paired differences are plotted in Figure 2.

### 5.2. Protocol-Matched Recent-Method Comparison

Under the common evaluation protocol, we ran a paired experiment on the current V2X-Radar-V conversion. All runs used the same 320/80 train/validation frames, five-channel single-frame radar input, car mapping, 160 epochs, spatial range, output budget, and independent double-precision ${\mathrm{AP}}_{40}$ evaluator. The adaptation adds radial-velocity Cartesian decomposition and pillar attention—the two RadarPillars design elements representable under the five-channel protocol. It is not the authors’ official multi-frame code; the common protocol does not include compensated velocity or timestamps.

Table 7 reports the paired V2X-Radar-V recent-method compatibility experiment under the common protocol.

The paired adaptation-minus-control differences have mean -1.8705 AP (sample SD 1.8377). The proposed strict route exceeds the adaptation in 3/3 paired seeds by +2.4473 AP on average (sample SD 1.5993). Control predictions are fresh inference from fixed formal checkpoints; all adapted rows are fresh training runs, and strict-route rows are the fixed final predictions evaluated by the same independent evaluator. The adaptation itself scores below the fixed-checkpoint PointPillars control in this evaluation (mean 39.6772 vs. 41.5476 AP), so it does not represent a strong recent-method baseline. This pilot therefore only checks whether the proposed gain survives one locally implemented recent-method design change; it is a compatibility check under the common protocol, not evidence that the proposed method is competitive with recent radar-only detectors evaluated under their own published protocols. This is a protocol-matched compatibility experiment, not a reproduction of the official RadarPillars implementation. The official RadarNeXt repository provides checkpoints, but its released VoD configuration uses seven-channel, five-frame inputs from data/vod/radar_5frames/; this is not the five-channel single-frame protocol used here. The official RADE-Net repository provides code for a K-Radar raw-tensor projection pipeline rather than a compatible compact point-cloud input. We therefore report both as protocol-incompatible methods and assign neither a surrogate point-input score. For reproducibility, we downloaded the public RadarNeXt source archive and confirmed that the released checkpoints include models/radarnext.pth and the VoD configuration projects/RadarNeXt/configs/radarnext.py. A full official VoD score still requires the released VoD five-frame data and the MMDetection3D environment; neither is part of the present five-channel single-frame evaluation package.

For the other locally available conversions, we ran matched-checkpoint routes on the locally complete Astyx validation conversion and the available K-Radar reduced validation conversion. These results use the same independent ${\mathrm{AP}}_{3D}$ R40 replay used for the formal ledger, but they are not official RadarPillars, RadarNeXt, or RADE-Net results.

Table 8 summarizes the locally reproducible external-data checks.

The corresponding prediction files, checkpoints, and evaluator outputs are provided with the reproducibility materials. These checks demonstrate executable external-data coverage, while the protocol-matched V2X-Radar-V experiment above remains the comparison used for the main claim.

For a public-checkpoint verification, we obtained the public RadarPillar checkpoint and completed inference on all three official VoD development/example frames available locally. The checkpoint loaded 139/139 parameters, and the official VoD evaluator running on a central processing unit (CPU) returned sample-level entire-area 3D AP values of 9.0909 (car), 14.1414 (pedestrian), and 12.1212 (cyclist), with a mean average precision (mAP) of 11.7845. Because the complete VoD validation release was not available locally, these three-frame values are reported only as reproducibility evidence and are not presented as a full benchmark score.

Table 9 gives the complete matched-seed evaluation for the strict route.

All paired differences are positive. Removing the largest gain does not change this result. The smallest margin is 0.1718 AP on V2X-Radar-V at seed 2027. The table note and experimental protocol give the remaining effect-size and uncertainty summaries.

### 5.3. Mechanism and Control Findings

We applied three fixed-parameter controls to the same fixed RDAR predictions. They use the same AP code and require neither expert predictions nor additional labels. In Table 10, standard box voting is positive for all three seeds on Astyx and V2X-Radar-V. Gaussian Soft-NMS and weighted box fusion do not meet this criterion on any dataset. None matches the strict route across all four datasets.

Across the 12 primary cells, vote-only changes the mean AP by −0.0274 and improves 6/12 cells. We then fixed one expert checkpoint within each dataset instead of matching the seed. Under this test, gate-only improves 11/12 cells, and gate-plus-vote improves 10/12. Both regressions occur on Astyx. The matched strict result is narrowest on V2X-Radar-V: its mean gain is 0.4216 AP, with two seed-level gains below 0.22 AP.

#### 5.3.1. Same-Expert Prediction-Level Comparison

This benchmark compares post-processing on identical RDAR and fixed-expert predictions. It holds one expert prediction stream constant within each dataset and varies the primary seed. It is distinct from the seed-matched, threshold-selected main endpoint. Frame alignment, car annotations, center-range evaluation, and the OpenPCDet-native float32 Compute Unified Device Architecture (CUDA) 40-point AP calculation at 3D IoU 0.50 are common across methods.

Pooled NMS uses confidence-descending oriented-3D suppression at IoU 0.50. The WBF adaptation uses equal model weights, greedy clusters above oriented 3D IoU 0.50, confidence-weighted centers/dimensions, and doubled-angle yaw averaging. Cluster confidence is the mean confidence multiplied by min(2,n)/2, where *n* is cluster size. Both fusion controls discard out-of-range centers before pooling and use at most 550 output boxes. The gate and voting operations retain their fixed candidate and residual handling; the final evaluator applies the same center-range filter to all outputs. The fixed-expert voting rule admits neighbors with scores no higher than the updated seed proposal score. Parameters were fixed for this calculation, without per-cell winner selection. This WBF variant is an explicit oriented-3D adaptation, not unmodified original WBF code.

Three additional controls preserve the primary box set exactly. Each non-residual primary proposal is matched to its highest-IoU expert proposal at the gate’s fixed IoU threshold of 0.30; unmatched proposals and the 50-entry residual tail remain unchanged. The controls replace a matched primary score by the arithmetic mean, maximum, or $\left(s_{p}+us_{e}\right)/\left(1+u\right)$, where *u* is match IoU. They do not add expert boxes, update coordinates, penalize unmatched proposals, or tune a mixing coefficient.

These results isolate access to a fixed pair of prediction streams more directly than the original single-stream controls, but they do not duplicate the main endpoint’s expert-selection protocol. Device/build sensitivity at an IoU threshold boundary is recorded in the implementation check. The primary TruckScenes seed-2026 prediction file gives 15.2100 AP (211 true positives) in the current CUDA implementation, versus 15.4127 AP (212 true positives) in the reference evaluation. All paired differences are calculated within the same evaluator and are reported separately from the seed-matched endpoint.

In this fixed-expert benchmark, pooled NMS gains 1.9691 macro AP and the WBF adaptation gains 2.3071, compared with 1.5732 for the gate and 1.5306 for gate-plus-vote. Pooled NMS and the gate each improve 11/12 cells; WBF and gate-plus-vote each improve 10/12. Thus, the proposed gate does not establish a general AP advantage over unrestricted two-stream ensembling. However, the strongest primary-box-preserving score control, IoU-weighted fusion, gains only 0.1860 macro AP and improves 6/12 cells. The gate exceeds this control in all 12 paired dataset–seed cells by 1.3872 AP on average (minimum 0.0310). Under the constraint that expert boxes cannot enter the output, the gate’s unmatched-proposal penalty and multiplicative support score therefore contribute beyond ordinary confidence mixing. The fair comparison supports this narrower mechanism claim while rejecting a broad superiority claim over WBF.

Why preserve the primary proposal set? Pooled NMS and the WBF adaptation gain more macro AP on average, but they do so by letting expert boxes enter the output directly, so a fraction of the reported detections are geometrically sourced from the second detector rather than from the primary stream. Three consequences follow: First, per-box provenance is lost—a downstream consumer (tracker, planner, or auditor) can no longer attribute each output box to a single, consistently trained detector, which complicates error attribution and safety review. Second, primary-stream output cardinality and box identity are not preserved across frames in the same way, which matters for trackers that associate detections by a stable primary identity rather than re-solving assignment every frame. Third, unrestricted fusion implicitly assumes that the two detectors’ score and geometry distributions are commensurable; the primary-box-preserving controls avoid this assumption because they never substitute expert geometry for primary geometry. The expert-consistency gate is therefore the appropriate choice when a deployment requires a single, traceable proposal stream with only its ranking and score informed by a second detector, whereas conventional two-detector fusion (pooled NMS, WBF) is preferable when maximizing average AP is the only objective and mixed-provenance output boxes are acceptable. We do not claim that the gate is preferable in general; Table 11 and this discussion report both the average-AP shortfall and the primary-box-preserving advantage so that this trade-off is explicit rather than implied by macro AP alone.

#### 5.3.2. Expert Complementarity and Shared Errors

At a fixed score threshold of 0.10, we perform greedy confidence-ordered, one-to-one matches to in-range car annotations at 3D IoU 0.50 independently for each detector. We record annotations detected by both, primary only, expert only, or neither. Expert-only coverage is a measure of potential additional object evidence, not the achieved recall of a realizable ensemble. We also compute the phi correlation between the two binary object-miss indicators and the Pearson correlation of per-frame false-positive counts. Scores of 0.30 and 0.50 are supplied as sensitivity checks in the accompanying 36-cell CSV, without choosing a favorable operating point.

Table 12 summarizes expert complementarity at the fixed score threshold of 0.10.

Expert-only detections demonstrate some nonidentical object coverage, while positive error correlations demonstrate shared failures. Separate training therefore supplies neither statistical independence nor a general reliability guarantee. These are descriptive checks on the evaluated conversions; repeated seeds and potentially related frames do not support independent-scene significance tests.

#### 5.3.3. Expert-Score Permutation Control and Proposal Changes

To test whether the gate gain can be reproduced by an arbitrary perturbation of expert confidence, we kept every expert box fixed and permuted its scores within each frame using a deterministic seed. The same gate and gate-plus-vote code was then evaluated on all four datasets and three primary seeds. The real expert scores produced +1.5732±1.1646 and +1.5306±1.3449 AP relative to the primary stream for gate and gate-plus-vote, respectively, with 11/12 and 10/12 positive cells. The score-permutation controls instead produced −1.6087±1.9436 and −1.6185±2.0870 AP, with only 3/12 and 2/12 positive cells. This is a negative control for the interpretation that the observed gain is explained by a generic random score perturbation; it does not prove that expert agreement is universally reliable.

The paired proposal check also confirms that the output count remains fixed at 550 boxes per frame, including the unchanged 50-box residual tail. On average, 28.58 of the first 500 primary proposals meet the expert IoU-0.30 match rule, while local voting changes the x,y,z,l,w,h or heading representation of 346.12 boxes per frame. These counts describe the operation of the archived implementation and are not additional accuracy endpoints.

#### 5.3.4. Progressive Construction

**Figure 3:**
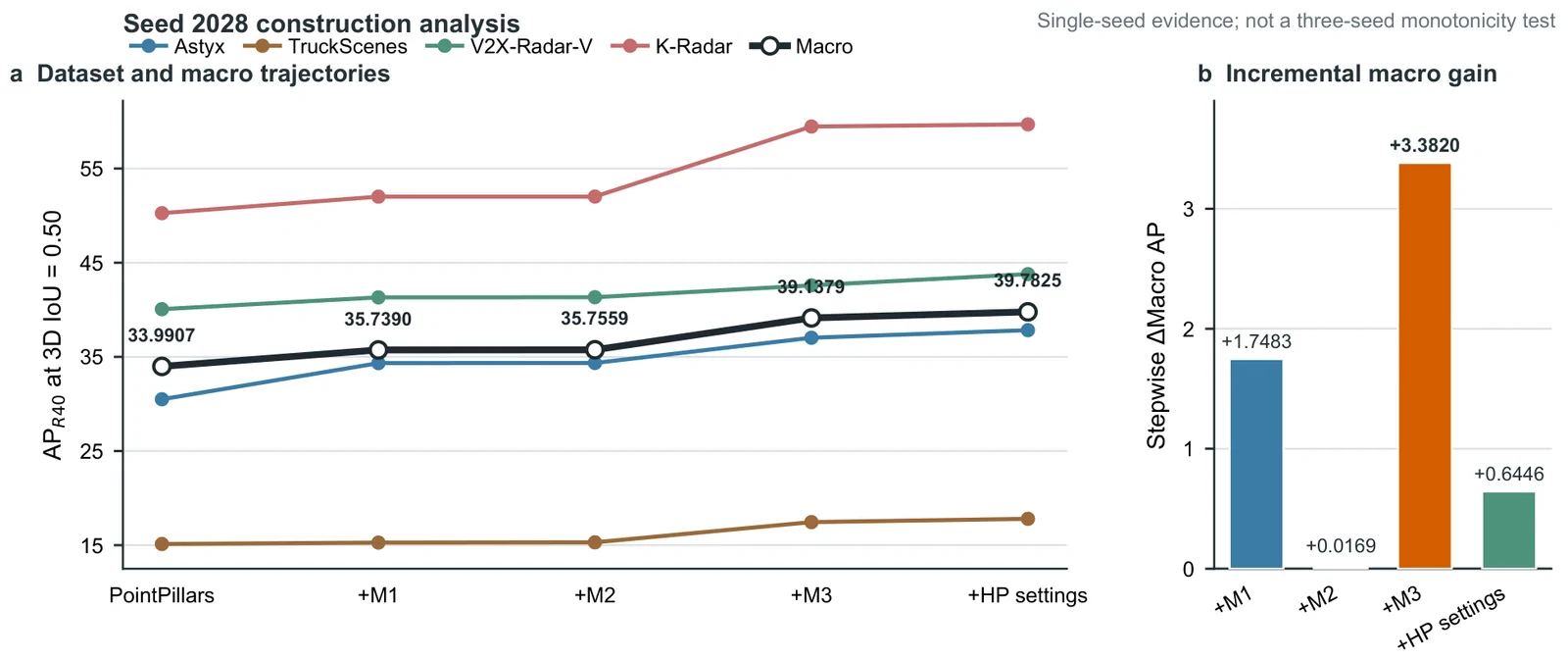
Progressive construction on seed 2028. M1 and M2 preserve and recover candidate evidence, while M3 supplies the dominant accuracy-oriented gain; the plot is a mechanism analysis for one seed rather than a multi-seed monotonicity claim. AP: average precision; RDAR: the candidate-preserving baseline.

Figure 3 follows the seed-2028 construction path on the four datasets. M1 has its clearest effect on Astyx, whereas M2 changes the result only slightly. M3 produces the largest macro step, especially on K-Radar. This visualization uses one seed; the three-seed analysis below reports stage means and sample SDs.

Relative to the preceding configuration, M1 adds 1.7483 macro AP and M2 adds 0.0169. M3 raises macro AP from 35.7559 to 39.1379. The corresponding gains are 2.6778 AP on Astyx, 2.1489 on TruckScenes, 1.2594 on V2X-Radar-V, and 7.4416 on K-Radar. The final accuracy-oriented adjustment adds 0.6446 AP.

#### 5.3.5. Three-Seed Progressive Analysis

To test whether the construction-path observation depends on seed 2028, we reassembled every available stage from the consistent result records for seeds 2026–2028. The first three rows come from the completed fair-ablation ledger; M3 is the qflr55 quality-alignment route in the separately frozen endpoint table. No values were reconstructed from the plotted curves. This analysis uses one version-consistent ledger throughout: its K-Radar PointPillars seed-2028 entry is 47.6224, whereas the seed-2028 construction figure in Figure 3 records 50.2659. We therefore do not mix that value into this table.

Table 13 reports the progressive analysis across all three seeds.

**Figure 4:**
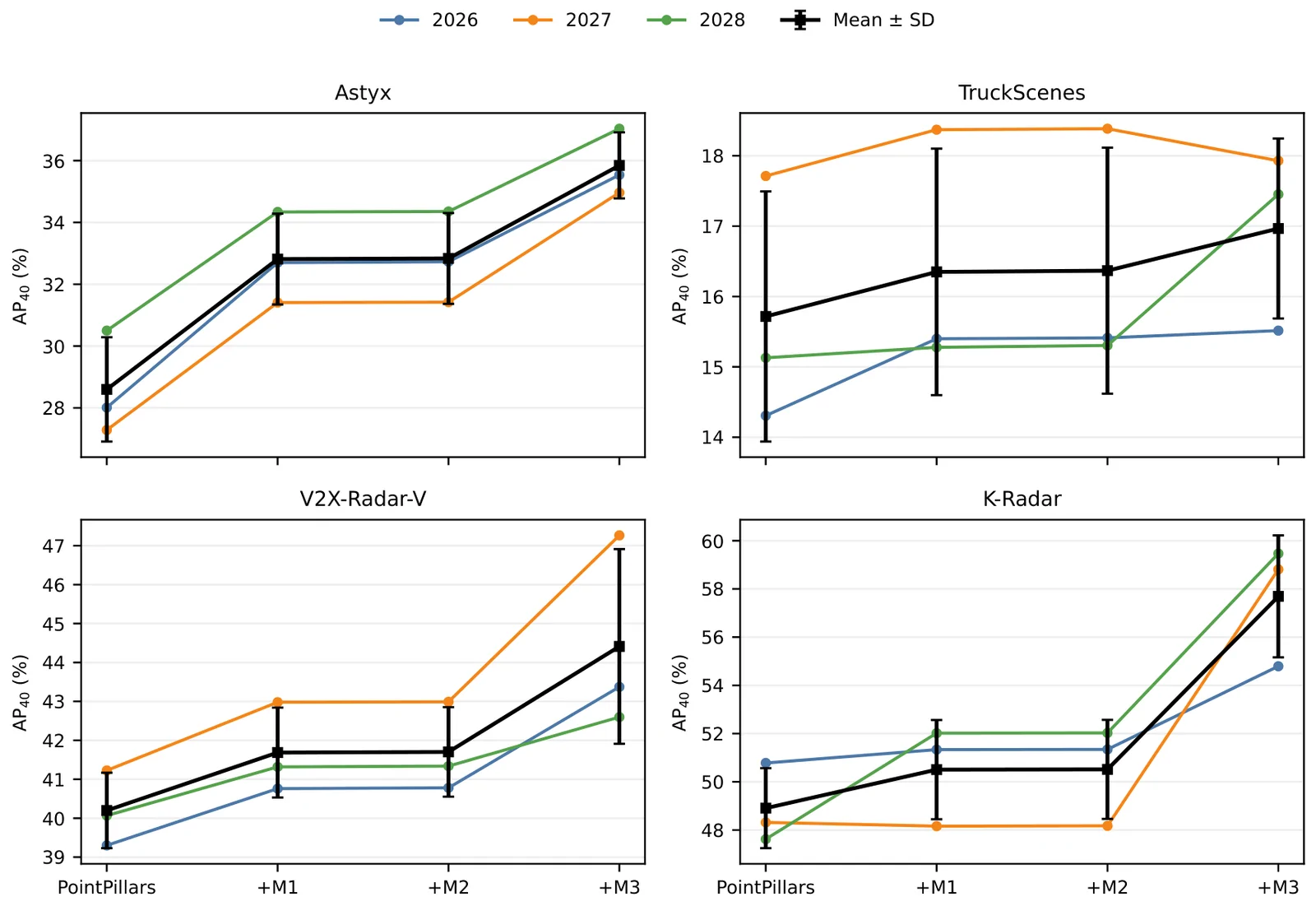
Seed-level trajectories and mean ± sample SD for the recovered four-stage progressive analysis. The figure reports all cells, including local stage regressions. AP: average precision; SD: standard deviation.

Figure 4 shows the corresponding seed-level trajectories and stage summaries.

Across all four datasets, macro AP rises from the PointPillars baseline to M3. The stage-wise pattern is not strictly monotone in every dataset–seed cell; accordingly, this result supports an average construction trend rather than a claim that each module independently improves every run.

As a paired statistical summary, we bootstrap the 12 dataset–seed cells. The PointPillars-to-M1 change is +1.9842 AP with a 95% cell-bootstrap interval of [+1.0576,+2.9753] and 11/12 positive cells; M1-to-M2 is +0.0159 with [+0.0124,+0.0197] and 12/12 positive cells; M2-to-M3 is +3.3730 with [+1.8709,+5.1423] and 11/12 positive cells. Sign-flip permutation p-values are 0.0015, 0.0005, and 0.0015, respectively. These statistics quantify the average construction trend only and do not imply that every module improves every individual cell.

### 5.4. Robustness, Calibration, and Threshold Transfer

**Figure 5:**
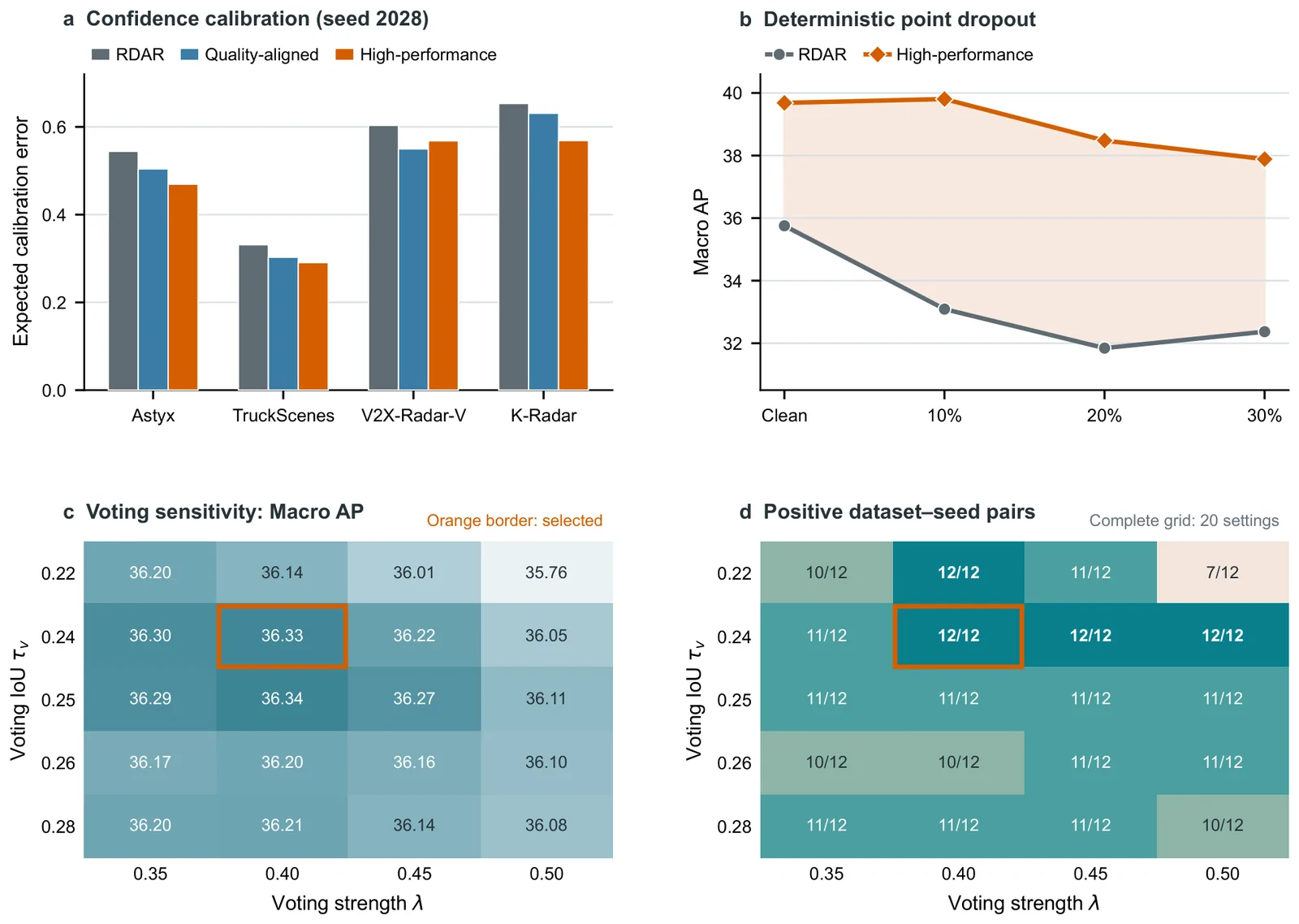
Diagnostic and boundary analyses. Panels compare calibration, controlled point loss, voting thresholds, and the number of positive paired cells across the reported routes. ECE: expected calibration error; AP: average precision.

Figure 5 summarizes calibration, controlled point loss, voting thresholds, and the number of positive paired cells.

ECE compares mean confidence with empirical correctness within confidence bins [36]. The high-performance route lowers ECE in all 12 three-seed comparisons, with a mean reduction of 0.0504. At seed 2028, the reductions are 0.0749 on Astyx, 0.0407 on TruckScenes, 0.0354 on V2X-Radar-V, and 0.0845 on K-Radar. M3-only gives the lowest ECE on V2X-Radar-V; the high-performance route is lowest on the other datasets. In contrast, the strict route increases ECE in all 12 comparisons by 0.0270 on average.

To complement that route-level diagnostic with a common detection-level definition, we also evaluate the fixed-expert benchmark using score-descending one-to-one matching. Predictions with scores of at least 0.10 are matched to the car ground truth at 3D IoU 0.50; ECE and adaptive ECE use 15 fixed bins, while Brier score and binary negative log-likelihood (NLL) are reported as proper scoring rules. The gate lowers mean ECE from 0.1143 to 0.1020, and gate-plus-voting gives 0.1029, but their Brier scores and NLLs are worse than those of the primary detector. Conversely, IoU-weighted score fusion gives the lowest Brier score and NLL despite worse ECE. These metric-dependent directions rule out a blanket calibration-improvement claim and expose the accuracy–score quality trade-off more fully.

Table 14 reports the detection-level calibration metrics in the fixed-expert benchmark.

We further tested calibration transfer by fitting one temperature on three datasets and evaluating it on the untouched fourth dataset, repeated in a leave-one-dataset-out manner. The transferred temperature reduces mean ECE-15 from 0.1020 to 0.0573, Brier from 0.1012 to 0.0969, and NLL from 0.3470 to 0.3216 over the 12 held-out cells. Eleven of twelve cells improve on all three metrics; the remaining K-Radar seed-2028 cell has a small Brier increase from 0.12938 to 0.12954. This is a calibration-transfer analysis, not a claim of uniform improvement by the uncalibrated gate.

**Figure 6:**
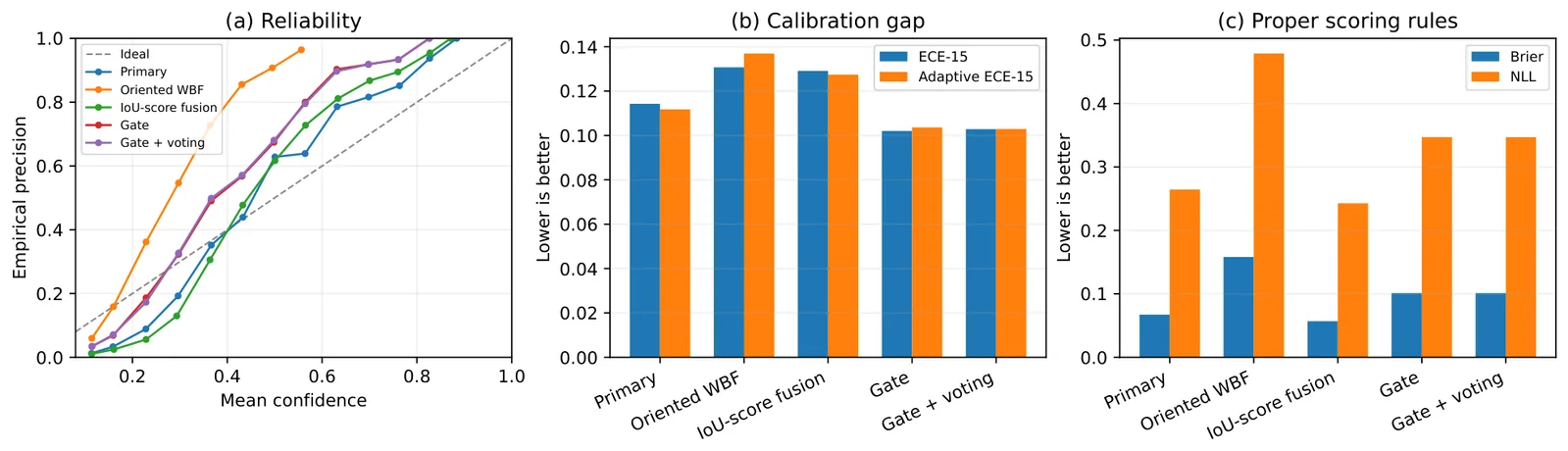
Detection-level calibration under identical fixed-expert prediction pairs, matching, and score filtering. Curves aggregate equal-width bin counts over all 12 dataset–seed cells; bars report cell-level means. ECE: expected calibration error.

Figure 6 shows the corresponding detection-level calibration curves and metric summaries.

The range-bin analysis gives the strict route 26 wins, 13 ties, and 9 losses. Its sparsity analysis gives 18 wins, 19 ties, 8 losses, and 3 missing cells. The high-performance route records 17 range-bin wins, 10 ties, and 21 losses. The bin-level counts include both improvements and regressions.

#### 5.4.1. Point-Dropout Stress Test

We dropped radar points before voxelization with a fixed seed of 3407. No model was retrained or adapted. The high-performance route remains ahead at 10%, 20%, and 30% dropout. Its changes from the clean checkpoint are +0.1242, −1.2030, and −1.7970 AP. The corresponding RDAR changes are −2.6651, −3.9114, and −3.3868—a mean difference in degradation of 2.3624 AP.

Across datasets and dropout rates, the high-performance route exceeds RDAR in 11/12 cells. The exception is TruckScenes at 30%: 11.9555 versus 12.0161 AP, a difference of −0.0606. Adding an exploratory vote fixes this cell but reduces the original result in 8/12 cells, so we leave the endpoint unchanged.

#### 5.4.2. Voting-Threshold Sensitivity

Figure 5c,d show the complete grid over voting IoU and strength. Three neighboring settings also remain positive in all 12 dataset–seed pairs: (0.22,0.40) reaches 36.1364 macro AP, (0.24,0.45) reaches 36.2165, and (0.24,0.50) reaches 36.0479. The highest grid value, 36.3393 at (0.25,0.40), is positive in 11 of 12 pairs. The selected (0.24,0.40) setting reaches 36.3278 macro AP and remains the best setting under the 12-of-12 criterion.

#### 5.4.3. Held-Out Threshold Transfer

For leave-one-dataset-out transfer, the conservative screen keeps settings with nine positive training differences and then selects the highest mean. The unconstrained screen selects by training mean alone. Without further tuning, the conservative rule gives positive held-out means for all four datasets and 11/12 positive seed differences. The unconstrained rule yields one negative cell: −0.2945 AP on V2X-Radar-V at seed 2027. This analysis is separate from the formal threshold-selected result. We also performed a stricter seed-holdout check: for each dataset, the candidate voting parameters were selected using seeds 2026 and 2027 only, without inspecting seed 2028, and then evaluated once on the untouched seed-2028 predictions. The selected settings produced positive held-out differences on all four datasets: +0.7313, +0.9136, +0.8367, and +1.3861 AP for Astyx, TruckScenes, V2X-Radar-V, and K-Radar, respectively. This four-test analysis strengthens the independence of threshold transfer, but its held-out unit is still one seed per dataset and should not be interpreted as a universal generalization guarantee.

Table 15 summarizes the leave-one-dataset-out threshold-transfer results.

Table 16 reports the independent seed-holdout threshold-transfer results.

**Figure 7:**
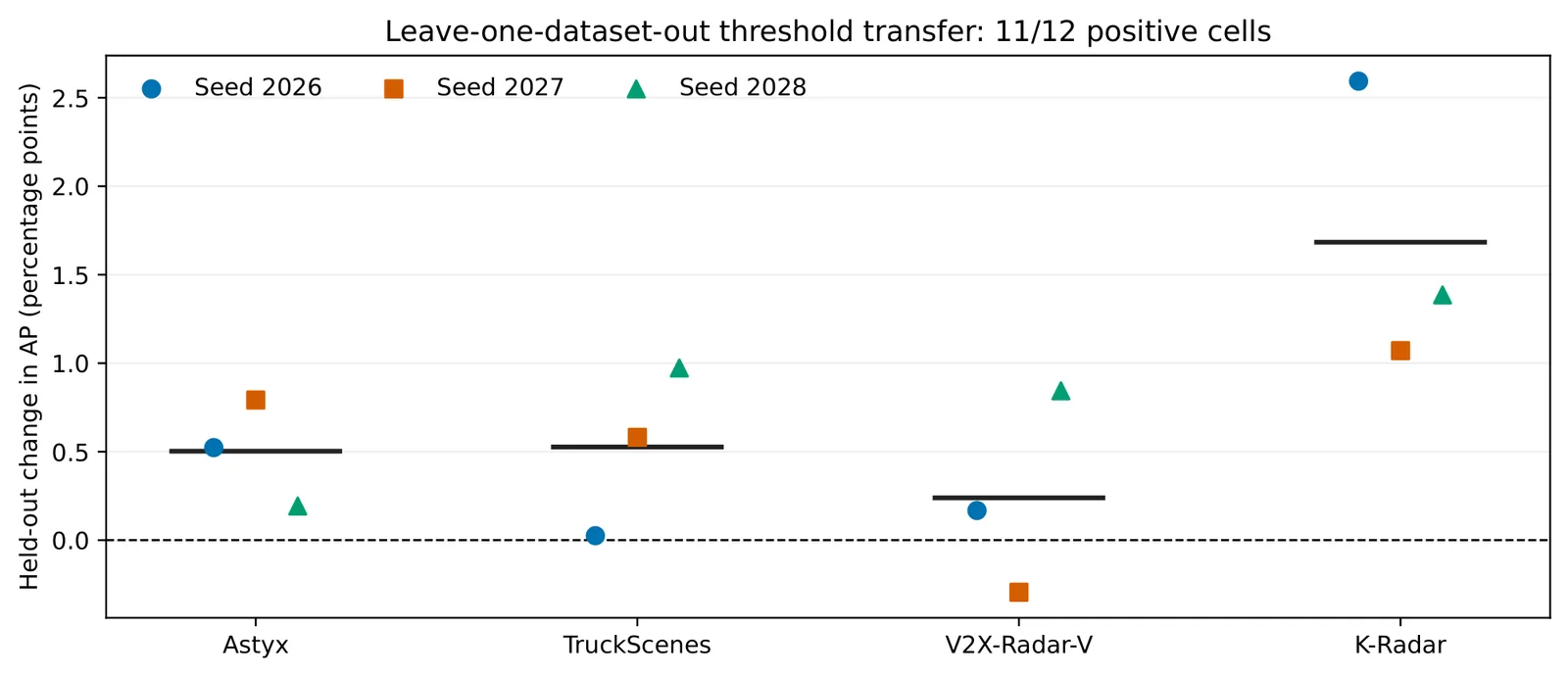
All twelve held-out differences. Horizontal black segments show dataset means; the dashed line denotes no improvement. The V2X-Radar-V seed-2027 decrease is shown explicitly. AP: average precision.

Figure 7 displays all twelve held-out differences from the leave-one-dataset-out analysis.

As a stricter parameter-transfer analysis, we also performed leave-one-cell-out parameter selection over the same 20-point voting grid. For each test cell, both parameters were selected using only the other 11 cells and then applied once to the untouched cell. The paired evaluation gives a mean held-out change of +1.4490±1.2614 AP, with 10/12 positive cells and a minimum of −1.2531 AP. The two negative cells are retained explicitly. This cell-level transfer analysis uses the deterministic CPU/Shapely evaluator for parameter-isolation analysis and does not replace the native CUDA main endpoint.

The two selection questions must be separated. The 12/12 result describes the configuration selected on the reported cells. The leave-one-dataset-out analysis excludes one dataset only when choosing among already-defined grid points; it does not establish that the architecture, candidate grid, or other settings were designed without exposure to that dataset. Its positive dataset means therefore support limited threshold transfer, while the negative held-out seed rules out a universal no-regression claim. A genuinely independent development/test separation remains a stronger prospective test.

### 5.5. Qualitative Successes and Failure Boundaries

**Figure 8:**
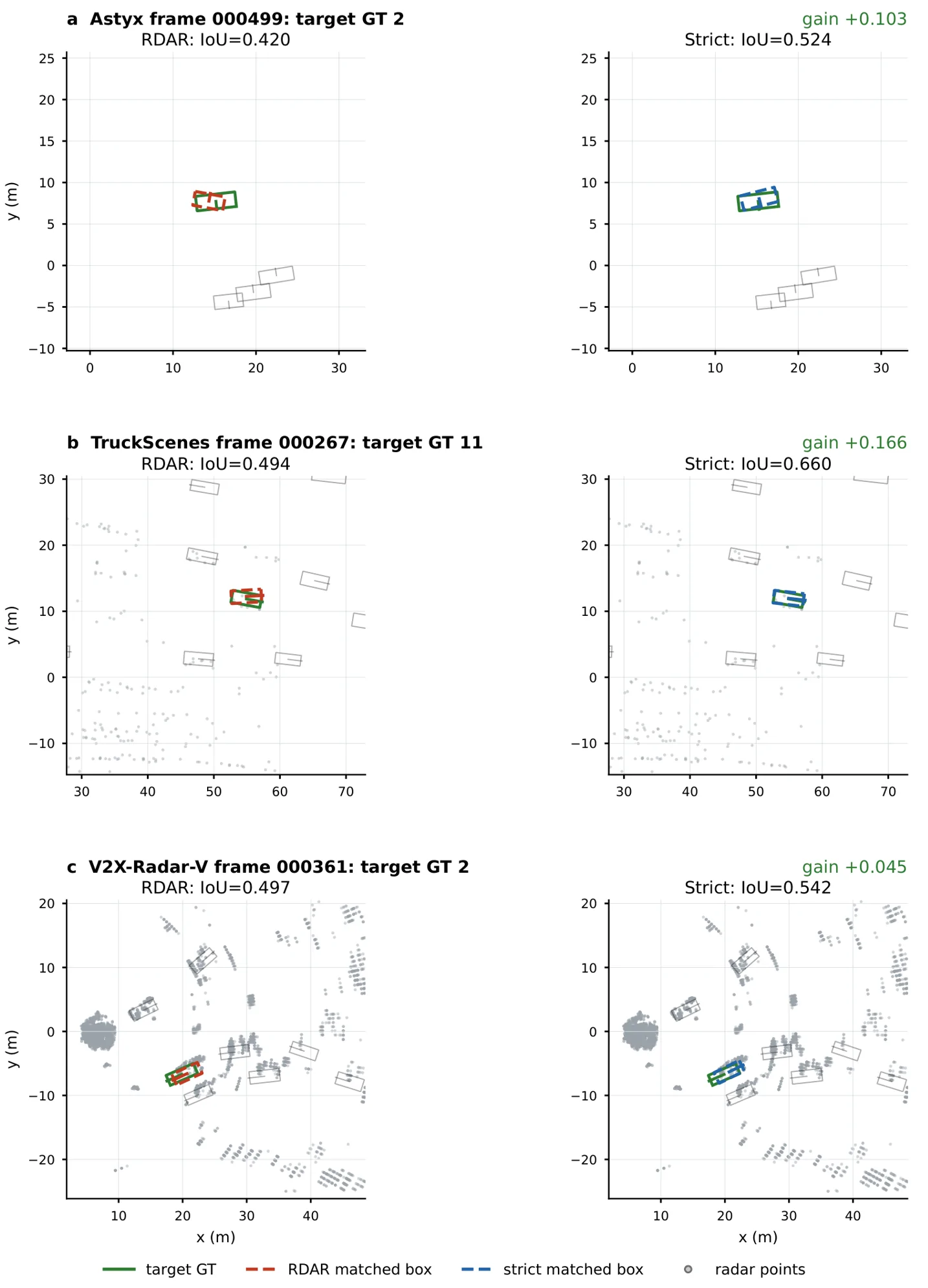
Qualitative bird’s-eye-view (BEV) cases comparing RDAR and the strict route against the same ground-truth targets. The constrained update changes center and heading while keeping the box dimensions fixed. RDAR: the candidate-preserving baseline.

Figure 8 compares RDAR and strict-route boxes for three selected borderline misses. The examples show how center and heading updates can cross the 0.50 matching threshold without changing the box dimensions. They do not indicate how often this occurs.

Conditional voting raises IoU from 0.4203 to 0.5235 in Astyx frame 000499. It raises IoU from 0.4940 to 0.6599 in TruckScenes frame 000267 and from 0.4970 to 0.5420 in V2X-Radar-V frame 000361. In each case, the restricted xy and heading update turns a borderline localization into a valid match without resizing the box.

#### Failure Cases and Geometry-Only Analysis

We analyze the paired predictions without retraining. For each in-range car annotation, we select the primary proposal with the highest pre-update 3D IoU among proposals with original RDAR score at least 0.10, excluding the final 50 residual proposals. We then measure the same proposal against the same annotation after voting. Selection is held fixed to isolate geometry from score re-ranking. Frame identifiers, proposal counts, unchanged heights and sizes, and unchanged residual boxes are checked before comparison.

Table 17 summarizes the selected-proposal geometry changes.

**Figure 9:**
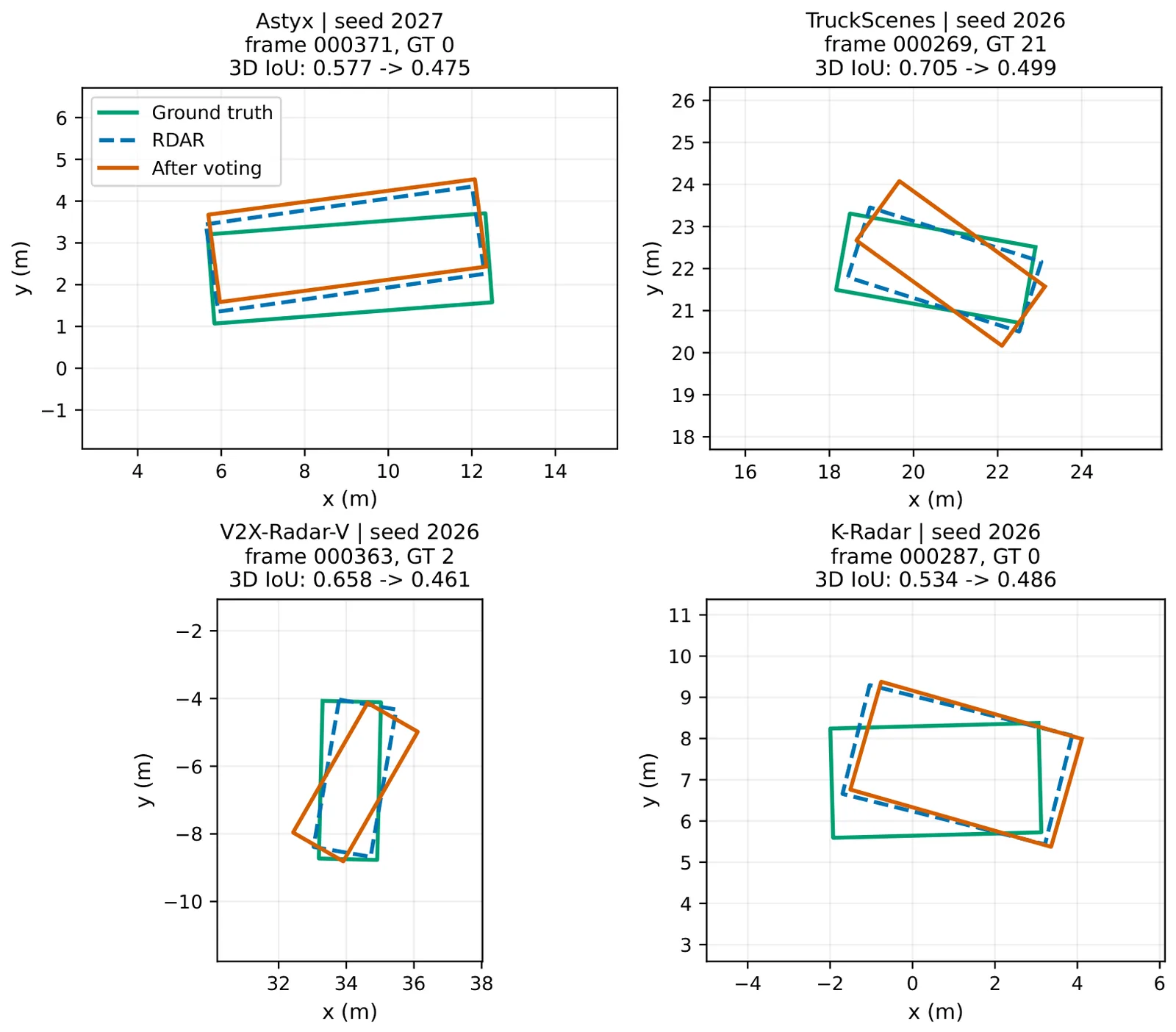
Deterministically selected failure cases from the paired predictions. For each dataset, a lost 0.50-IoU crossing is prioritized, followed by the largest IoU decrease; seed, frame, and annotation index break ties. The plots show BEV box footprints, while labels report full 3D IoU. These examples are intentionally adverse cases, not an estimate of typical detection performance. BEV: bird’s-eye view; IoU: intersection-over-union; 3D: three-dimensional.

Figure 9 illustrates the deterministically selected failure cases.

Local agreement does not ensure alignment with the annotated object. A weighted center or heading update can therefore worsen localization or turn a borderline geometric match into a miss even when aggregate AP improves. This analysis measures selected-proposal geometry only: it does not include all effects of re-ranking, duplicate suppression, or one-to-one AP matching, and it does not establish a causal role for point density or occlusion.

Across 3174 eligible selected-proposal observations, 1558 (49.1%) improved in 3D IoU, 1604 (50.5%) decreased, and 12 were unchanged. There were 69 lost and 79 gained 0.50-IoU crossings. The mean IoU change was +0.00026. These counts quantify the fixed selected-proposal diagnostic; they are not AP true/false-positive counts or an estimate of unseen-scene failure probability.

### 5.6. Computational Implications

Table 18 reports parameter counts and timing measurements. M3 changes the training target rather than network width, so the M3-only and high-performance detectors retain the RDAR parameter count. Voting adds no trainable parameters, but it adds proposal-refinement time. We report detector forward time, isolated voting time, and endpoint time separately.

To complement the RTX 3090 component profile, we evaluated loaded epoch-160 weights on an RTX 4060 laptop GPU. The primary and independently trained expert were timed both separately and sequentially in one process. We also evaluated the vectorized implementation on all 1020 dataset–seed frames and verified its numerical output against the original implementation before treating it as equivalent.

Table 19 reports the loaded-weight detector timing analysis.

Both M3-based detectors have 4.830 M parameters, the same as RDAR. The expert used by the strict route has 4.868 M parameters, and voting has no trainable weights. On the RTX 3090, the high-performance detector remains below 9 ms per frame but is slower than RDAR. The isolated 500-proposal voting loop takes about 22–23 ms. It constructs 3D IoUs on the GPU and then visits the local neighborhoods in a Python loop. Python 3.10 is recommended for reproduction; the interpreter version used for the original timing measurements could not be recovered from the available experiment records.

In the loaded-weight batch-one timing analysis, the primary and expert require 6.554±1.162 and 6.591±1.133 ms, respectively. Running both models sequentially in one process takes 13.106±1.621 ms (median 12.582 ms; P95 16.326 ms) with 211.841 MiB peak allocated memory. This directly measured two-detector row excludes gate/vote computation. Across all 1020 archived frames, the vectorized gate/vote outputs differ from the original implementation by at most $1.44\times 10^{-7}$ over the evaluated score, coordinate, and wrapped-yaw quantities; the scientific thresholds and outputs are therefore preserved to floating-point tolerance.

A separate batch-1 profile measures primary forward, expert forward, and post-processing in one process on an RTX 4080 SUPER (NVIDIA Corporation, Santa Clara, CA, USA). The mean times are 16.08 ms on Astyx, 23.11 ms on TruckScenes, and 21.62 ms on V2X-Radar-V. These values correspond to 62.2, 43.3, and 46.3 Hz, with 197–201 MB peak allocated memory. The sampled Astyx frames return 20–27 proposals, unlike the 500-proposal RTX 3090 component profile, so the two sets of timings are not combined. In a separate scaling test, voting takes 1.5–1.6 ms for 50 proposals and 9.7–10.1 ms for 500. The peak incremental allocation is approximately 6.7 MB.

## 6. Discussion

No single route dominates across all criteria. The high-performance route has the larger mean AP gain and favorable route-level ECE, but one matched comparison is negative. The strict route improves all 12 threshold-selection dataset–seed cells, with a smaller mean gain and mixed calibration behavior. The observed trade-off is between higher average detection quality and fewer regressions in the selected evaluation cells, not a guarantee that either route avoids regressions after deployment.

### 6.1. Mechanism and Relation to Prior Work

Candidate preservation leaves alternative hypotheses available after the first suppression step, while residual recovery restores proposals that would otherwise disappear. In both the original seed-2028 screen and the consolidated three-seed analysis, most of the macro-AP increase comes from M3. Across the three-seed evaluation, macro AP changes from 33.3551±0.2683 for PointPillars to 35.3394±0.3577 after M1, 35.3552±0.3581 after M2, and 38.7283±1.2692 after M3. Localization-aware ranking is therefore the main average contributor in this chain, although local stage regressions prevent a per-run monotonicity claim.

The controls help explain why the strict route behaves differently from generic post-processing. Vote-only improves 6 of the 12 primary cells. Neither fixed Soft-NMS nor weighted box fusion produces three positive seeds on any dataset. When one expert checkpoint is fixed within each dataset, gate-only is positive in 11/12 cells and gate-plus-vote in 10/12. These results place most of the observed benefit in the expert check, with voting playing a more selective role. The same-expert comparison resolves the most direct additional-detector confound. Pooled NMS and WBF obtain larger macro gains when expert boxes may enter the output, so the proposed route is not the strongest unrestricted ensemble. Under a primary-box-preserving constraint, however, the best ordinary score-fusion control gains 0.1860 macro AP in 6/12 cells, compared with 1.5732 and 11/12 for the gate. The gate is higher than this control in all 12 paired cells, by 1.3872 AP on average. This supports the narrower claim that unmatched-proposal penalization and multiplicative expert support do more than mix two confidence values while retaining the primary candidate set.

The expert check also separates the strict route from existing post-processing methods. Soft-NMS adjusts scores from proposal overlap, whereas weighted box fusion averages coordinates using detector confidence [13,30]. Here, an independently trained expert provides cross-model support for re-ranking; local geometric agreement then determines the voting neighborhood. Unlike pooled NMS and WBF, gate-only does not insert expert boxes or average primary and expert coordinates. This output constraint, rather than universal ensemble superiority, is the meaningful distinction supported by the fair comparison. Separate training does not imply statistically independent errors, since both models see the same radar frame and can share localization failures. M3 acts earlier by aligning the ranking score with localization quality, an idea shared with IoU-aware confidence methods [14,34]. Our experiments examine how these two operations interact on a fixed PointPillars-style backbone; they do not show that either class of method can be replaced.

### 6.2. Operational Trade-Offs and Boundaries

Calibration conclusions depend on both the route and the scoring rule. In the archived route-level comparison, the high-performance route lowers ECE in all 12 cells, whereas the strict route raises it in all 12. In the new fixed-expert one-to-one detection analysis, however, the quality gate lowers 15-bin ECE in 10/12 cells (mean 0.1143 to 0.1020), while worsening Brier score and binary NLL in all 12; IoU-aware score fusion obtains the best mean Brier score and NLL. Thus, no method supports a blanket calibrated-probability claim, and scores should be recalibrated before risk-sensitive thresholding. The high-performance route is the more natural choice when ranking and confidence quality matter together.

Robustness is similarly uneven. The range and sparsity analyses contain both wins and losses, and TruckScenes regresses at 30% point dropout. Point density, return geometry, or the localization errors presented to the expert may account for some of this variation, but the current analyses do not separate these factors. The gains are consequently specific to the tested datasets and operating regimes.

The computational profiles add another practical constraint. The high-performance detector retains the RDAR parameter count but has higher forward latency. In the loaded-weight timing analysis, sequential primary–expert inference takes 13.106 ms on average and 16.326 ms at P95 on the RTX 4060 Laptop GPU, before gate/vote computation. The strict route also requires expert inference, and its 500-proposal Python voting loop takes about 22–23 ms on the RTX 3090. Because the exploratory RTX 4080 SUPER profile uses different proposal counts, it is not directly comparable with the component-level RTX 3090 measurements. A deployment decision must account for calibration, the cost of a regression, and the available latency budget.

An additional proposal-budget analysis provides a practical operating point. Across the twelve dataset–seed cells, reducing the retained budget from 550 to 100 changed the gate-plus-voting macro AP from 36.8689 to 36.7852, while the CPU/Shapely timing per frame decreased from 320.4 ms to 14.3 ms; budgets of 200, 300, and 400 required 59.3, 120.7, and 189.3 ms, respectively. These timings are relative CPU timing measurements rather than GPU deployment claims. At the full budget, the corresponding stratified gain over the primary route was 1.61 AP points for near-range targets, 0.50 for mid-range targets, and 0.23 for far-range targets; the gain was 1.64 points in sparse frames and 1.61 in dense frames. Thus, the method is most useful for close and moderately observable targets, whereas long-range performance remains limited by the underlying radar evidence. Because the study uses offline benchmark conversions, this analysis is a practical sensitivity analysis rather than a real-world deployment demonstration.

### 6.3. Limitations and Future Work

The conclusions are limited to radar-only car detection, four dataset conversions, three seeds, and one PointPillars-style detector. The smallest strict gain is 0.1718 AP, and the voting threshold is selected within the same evaluation protocol. Leave-one-dataset-out selection reduces this dependence but still produces one negative held-out seed. The frame manifests do not allow a scene-disjoint evaluation. The original progressive figure uses one seed; the three-seed analysis is computed from consistent result records rather than a new scene-disjoint experiment, and one K-Radar baseline version difference is reported instead of being silently pooled. The original qualitative examples show successful corrections and are therefore not representative of all cases. The deterministic failure analysis also exposes harmful geometric updates, but neither set of selected examples estimates performance on unseen scenes.

A remaining limitation is the absence of a protocol-matched comparison with recent public radar-only detectors. Published scores based on different dataset conversions, spatial ranges, and evaluators cannot be inserted fairly into the present table. Future work should focus on development-set threshold selection, scene-aware splits, additional detector families and classes, and reproduced public baselines under the same evaluator. Post hoc calibration of the strict route and a batched or sparse-neighborhood voting implementation would address the two main deployment costs identified here.

## 7. Conclusions

We introduced a proposal-level refinement framework for radar-only 3D car detection that leaves the detector backbone unchanged. It retains alternative candidates, uses localization quality in proposal ranking, and checks proposed updates against an independently trained expert. Over four dataset conversions and three seeds, the strict route improved AP in all 12 matched dataset–seed comparisons under the threshold-selected evaluation protocol, with an average gain of 0.9726 AP. Leave-one-dataset-out threshold selection retained positive means on all four datasets but not positive gains in every seed. The high-performance route achieved the larger macro-AP increase, from 35.3552 to 39.1214, and lowered ECE in every comparison, but it regressed on one TruckScenes seed. This larger gain comes from the M3 localization-quality-aware confidence head and relaxed positive assignment; the high-performance route does not use the expert-consistency gate, so the gain should not be attributed to the expert-consistency mechanism emphasized in the title.

Neither route was better on every criterion. The strict route was more consistent in matched AP, but its archived route-level ECE increased in all 12 comparisons, and it added the cost of expert inference and local voting. The separate fixed-expert analysis found lower mean ECE but worse Brier score and NLL for the quality gate, confirming that calibration depends on the metric and matching protocol. The high-performance route was less consistent across seeds, although it gave stronger average accuracy and favorable route-level ECE. These findings are confined to radar-only car detection with four dataset conversions, three seeds, and a PointPillars-style backbone. Further evaluation should choose thresholds on a development set and reproduce recent methods under the same evaluator. Tests on other detectors and classes, together with a faster voting implementation, would show how well the findings carry beyond the current setting.

## Figures and Tables

**Table 1:** Definitions of the operating routes and controls. M1 and M2 form the shared candidate-preserving proposal stream; the remaining columns identify the additional scoring or refinement operations. M1: candidate-preserving non-maximum suppression; M2: residual proposal recovery; M3: localization-quality-aware confidence; RDAR: the M1+M2 baseline; IoU: intersection-over-union; AP: average precision.

Configuration	M1 + M2 Stream	M3 Score	Expert Gate	Local Vote	Selection Objective
RDAR baseline	Yes	Binary objectness	No	No	Reference
M3-only control	Yes	IoU-aware quality	No	No	Isolate quality ranking
High-performance route	Yes	IoU-aware quality + relaxed assignment	No	No	Maximize macro AP
Strict route	Yes	RDAR score	Yes	Yes	Positive paired differences

**Table 2:** Conceptual comparison of proposal operations. “Independent evidence” means an additional separately trained prediction stream, not statistical independence of its errors. WBF can combine several models; its single-stream control in this study does not use the expert. NMS: non-maximum suppression; IoU: intersection-over-union; WBF: weighted box fusion.

Operation	Input	Additional Evidence	Score Operation	Geometry Operation
Soft-NMS [13]	Decoded boxes and scores	Not required	Overlap-dependent score decay	No coordinate averaging
IoU-aware confidence [14,34]	Detector features/quality targets	No second detector required	Learn localization-sensitive ranking	Quality scoring itself need not move boxes
Standard box voting	Seed boxes and neighbors	Not required	Scores weight votes; rule-dependent rescoring	Average supported coordinates
Weighted box fusion [30]	One or multiple box streams	Optional multiple models	Aggregate cluster confidence	Fuse clustered coordinates
Expert gate (strict route)	Primary boxes plus radar expert	Yes, a second radar detector	Confidence and overlap product, or unmatched penalty	None at gate stage
Local voting after gate	Primary neighborhoods and gated scores	Via gate-derived weights	Preserve gated output scores	Interpolate x,y, yaw; preserve z,l,w,h

**Table 3:** Principal notation and domains. Indices and weights are local to the indicated operation. All overlaps used here are oriented 3D IoUs.

Symbol	Definition
$X,N_{x},x_{n}$	Radar point set, its cardinality, and a point with spatial and radar attributes.
$P,N_{p},b_{i},s_{i},c_{i}$	Primary proposal set, count, oriented box, confidence in [0,1], and class. Box centers/dimensions are in meters; yaw is in radians.
$\pi ,\tau_{\mathrm{nms}}$	Score-descending ordering and suppression-overlap threshold.
$R,r_{j},t_{j},K_{r}$	Residual proposals, their boxes and scores, and retained count; $i^{⋆}$ is the best primary match for a residual.
$s_{\min}^{+},t_{\max},\epsilon$	Minimum positive primary score over the evaluated prediction collection, per-frame maximum residual score, and denominator protection $10^{-12}$. The collection-level minimum defines a batch prediction operation, not a separately validated online estimator.
$g_{i},q_{i},{\hat{q}}_{i}$	Assigned ground-truth box, detached localization IoU, and quality-adjusted positive target.
ρ,γ,β	Positive-target floor, IoU-target exponent, and quality-focal modulation exponent.
$\ell_{i},p_{i},C,N_{+}$	Classification logit (distinct from box center $z_{i}$), sigmoid probability, non-ignored anchors, and positive-anchor normalization count. Implementations clamp this count to at least one.
$E,e_{j},u_{j},j^{⋆}\left(i\right),v_{i}$	Expert predictions, expert box/score, best expert index for primary *i*, and maximum expert IoU. Empty expert sets follow the unmatched rule.
$\alpha ,\eta ,\kappa ,s_{i}^{\prime }$	Expert-confidence exponent, overlap exponent, unmatched penalty and gated confidence. The matched-score implementation clips confidence bases to $\left[10^{-8},1\right]$.
$N_{i},w_{j},c_{i},\lambda$	Voting neighbors, weight $\max(s_{j}^{\prime },10^{-8})$, consensus geometry, and interpolation strength. In residual fusion only, the two weights are the primary/residual confidences instead.
$f_{i},c_{i}^{\mathrm{pg}},d_{i},a_{i}$	Normalized pillar feature, within-sample mean pillar context, normalized point count, and pillar-gate activation. These are distinct from classification probability $p_{i}$ and voting consensus $c_{i}$.
σ,GAP,⊙,[;]	Sigmoid, global average pooling, element-wise multiplication, and feature concatenation; *g* and *h* denote the channel-gate and context mappings described in the expert architecture.

**Table 4:** Reported settings and the displayed voting search grid. “Reported setting” specifies the frozen implementation; it does not assert that the parameter was never explored during development. The 20-point displayed grid is not an exhaustive history of all exploratory runs.

Parameter	Documented Range/Alternatives	Selected Value	Criterion or Role
M1 score/NMS IoU	Baseline (0.10,0.01); preserving (0,0.50)	(0,0.50)	Reported candidate-preservation setting
NMS budgets	Reported setting	4096/500	Pre-/post-NMS maxima
Residual $K_{r},\tau_{r}$	Reported setting	50,0.10	Residual count and primary-match threshold
Quality ρ,γ,β	Reported setting	0.55,1,2	Positive floor, IoU exponent, focal exponent
Gate $\tau_{e},\alpha ,\eta ,\kappa$	Reported setting	0.30,0.30,0.25,0.50	Expert support and score weighting
Voting $\tau_{v}$	{0.22,0.24,0.25,0.26,0.28}	0.24	Jointly selected with strength
Voting λ	{0.35,0.40,0.45,0.50}	0.40	Highest macro AP among 20-grid settings positive in all 12 cells
Voting geometry	Reported setting	x,y,θ only	Preserve vertical center and dimensions
Evaluation IoU/recall	Common protocol	0.50/40 levels	Same metric for every reported method
Training seeds/epochs	Common protocol	2026–2028/160	Matched final-checkpoint comparisons

**Table 5:** Dataset scope and common protocol for the formal four-dataset, three-seed evaluation.

Dataset	Fixed Conversion	Platform or Acquisition Setting	Model Input	Spatial Range [x,y,z] (m)	Formal Seeds
Astyx	Astyx	Automotive passenger-vehicle scenes	Radar point cloud	[0,60]×[−25,25]×[−3,2]	2026, 2027, 2028
MAN TruckScenes	TruckScenes-mini	Autonomous trucking scenes	Radar point cloud	[0,60]×[−25,25]×[−3,2]	2026, 2027, 2028
V2X-Radar-V	V2X-Radar-V-400	Vehicle-side cooperative-perception subset	4D radar point cloud	[0,60]×[−25,25]×[−3,2]	2026, 2027, 2028
K-Radar	K-Radar-400	Driving scenes under varied weather conditions	4D radar point cloud	[0,60]×[−25,25]×[−3,2]	2026, 2027, 2028

All rows use the paper’s fixed car-class mapping and are evaluated with ${\mathrm{AP}}_{R40}$ at a 3D-IoU match threshold of 0.50. Each method is trained independently for the three listed seeds, and paired method–RDAR differences are calculated within the same dataset and seed. The spatial range is the common model input range recorded in the fixed configurations, not a claim about the physical maximum range of each sensor.

**Table 6:** Main three-seed comparison of RDAR, the M3-only control, the high-performance route, and the strict route. AP: average precision; RDAR: the candidate-preserving baseline; M3: localization-quality-aware confidence.

Route	Astyx	TruckScenes	V2X-Radar-V	K-Radar	Macro	ΔMacro	Positive Paired Cells
RDAR	32.8347±1.4689	16.3671±1.7480	41.7029±1.1490	50.5163±2.0546	35.3552	–	–
M3-only	35.8438±1.0680	16.9661±1.2785	44.4122±2.5011	57.6910±2.5304	38.7283	+3.3731	11/12
High-performance	36.9801±0.9748	16.8063±1.1764	44.3786±0.5122	58.3208±1.6943	39.1214	+3.7662	11/12
Strict	33.6898±1.7215	17.2878±1.7127	42.1245±1.0852	52.2091±2.5654	36.3278	+0.9726	12/12

Values are ${\mathrm{AP}}_{R40}$ mean ± sample standard deviation over seeds 2026, 2027, and 2028 at a 3D-IoU threshold of 0.50. Macro is the unweighted mean of the four dataset means, and ΔMacro is measured from RDAR. Bold values mark the largest result in each dataset or aggregate column. “Positive paired cells” counts the dataset–seed cells with an AP increase relative to RDAR. The M3-only control and high-performance route correspond to the fixed quality-aware and accuracy-oriented configurations described above.

**Table 7:** Paired V2X-Radar-V recent-method compatibility experiment under the common protocol. These are locally evaluated predictions, not published scores copied across protocols. The standard deviation (SD) is across seeds 2026–2028. AP: average precision.

Method	Seed/Statistic	Validation Frames	${\mathrm{AP}}_{40}$
PointPillars (fixed-checkpoint control)	2026	80	40.9544
RadarPillars-inspired adaptation	2026	80	39.8200
Proposed strict route	2026	80	40.9969
PointPillars (fixed-checkpoint control)	2027	80	45.2021
RadarPillars-inspired adaptation	2027	80	41.2400
Proposed strict route	2027	80	43.1617
PointPillars (fixed-checkpoint control)	2028	80	38.4864
RadarPillars-inspired adaptation	2028	80	37.9715
Proposed strict route	2028	80	42.2148
PointPillars (fixed-checkpoint control)	Mean ± SD	240	41.5476±3.3969
RadarPillars-inspired adaptation	Mean ± SD	240	39.6772±1.6389
Proposed strict route	Mean ± SD	240	42.1245±1.0852

**Table 8:** Locally reproducible external-data checks. AP: average precision; 3D: three-dimensional; R40: the 40-recall-position evaluation.

Dataset	Route	Validation Frames	${\mathrm{AP}}_{3D}$ R40
Astyx	qflr55 checkpoint	100	29.2455
Astyx	q55rpa50-kprior checkpoint	100	35.6454
K-Radar reduced	fair PointPillars checkpoint	80	47.6431

**Table 9:** Complete matched-seed reliability evaluation for the strict route. AP: average precision; RDAR: the candidate-preserving baseline.

Dataset	Seed	RDAR AP	Strict AP	ΔAP
Astyx	2026	32.7281	33.5503	+0.8222
Astyx	2027	31.4220	32.0422	+0.6202
Astyx	2028	34.3540	35.4768	+1.1228
TruckScenes	2026	15.4127	15.8892	+0.4765
TruckScenes	2027	18.3845	19.1980	+0.8135
TruckScenes	2028	15.3041	16.7762	+1.4721
V2X-Radar-V	2026	40.7802	40.9969	+0.2167
V2X-Radar-V	2027	42.9899	43.1617	+0.1718
V2X-Radar-V	2028	41.3385	42.2148	+0.8763
K-Radar	2026	51.3450	53.9510	+2.6060
K-Radar	2027	48.1767	49.2631	+1.0864
K-Radar	2028	52.0271	53.4132	+1.3861

Strict AP is the RDAR value plus the fixed matched difference for the strict route. Across the 12 cells, the mean, median, minimum, and maximum ΔAP are 0.9726, 0.8493, 0.1718, and 2.6060, respectively. The nonparametric bootstrap 95% interval for the mean is [0.6468, 1.3536]. The exact sign test gives one-sided p=0.000244 and two-sided p=0.000488; these tests are auxiliary summaries for the finite evaluated cells. They do not correct for selecting the voting configuration on these same cells, and the cells are not twelve independent datasets. The reported probabilities and interval must not be interpreted as selection-adjusted evidence of out-of-sample consistency.

**Table 10:** Three-seed controls for generic proposal post-processing. Values are mean AP differences relative to RDAR. AP: average precision; RDAR: the candidate-preserving baseline.

Control	Astyx	TruckScenes	V2X-Radar-V	K-Radar	Datasets Positivein All Three Seeds
Standard box voting	+0.4723	−0.0831	+0.5342	+0.0097	2/4
Gaussian Soft-NMS (σ=0.5)	−0.1831	+0.0909	−0.2098	+0.0196	0/4
Weighted box fusion (IoU =0.5)	−0.0118	−0.1305	−0.2474	−0.1896	0/4
Strict route	+0.8551	+0.9207	+0.4216	+1.6928	4/4

All controls start from the same fixed RDAR prediction files and evaluator; control parameters were fixed before three-seed aggregation. These controls use one prediction stream: the WBF row is not a two-detector ensemble using the strict route’s expert. The strict route has access to additional expert predictions, so this table alone cannot isolate its mechanism from that added source of evidence.

**Table 11:** Same-expert prediction-level comparison using a common OpenPCDet-native float32 CUDA evaluator. Mean ± sample SD is over primary seeds; the fixed expert is the same prediction file for all three seeds and is therefore shown once without an artificial seed SD. All rows are reported, including controls that outperform the gate. SD: standard deviation; CUDA: Compute Unified Device Architecture; AP: average precision.

Method	Astyx	TruckScenes	V2X-Radar-V	K-Radar	Macro
Primary RDAR	32.8347±1.4689	16.2996±1.8063	41.7029±1.1491	50.5162±2.0546	35.3383
Fixed expert	25.9046	20.4147	41.5628	47.3941	33.8191
Pooled 3D NMS	33.0255±1.2458	20.5253±0.9769	43.1995±0.9096	52.4795±1.6268	37.3075
Oriented-3D WBF adaptation	32.6375±0.7771	22.1176±2.0250	44.8255±0.3754	51.0013±2.3674	37.6455
Mean-score fusion	29.7855±0.5898	16.4268±1.6689	37.2130±1.5090	44.2476±1.4126	31.9182
Max-score fusion	13.1852±0.3678	7.6783±0.1477	14.3839±0.4861	18.8982±0.9621	13.5364
IoU-weighted score fusion	32.6367±1.1224	17.8837±1.8389	41.5248±1.5052	50.0519±1.5044	35.5243
Expert gate	32.8994±0.9730	19.3528±1.5905	43.0075±1.4623	52.3865±2.3029	36.9115
Gate + local voting	32.7154±0.2950	18.8800±1.5335	43.2306±1.3063	52.6496±2.0979	36.8689

**Table 12:** Expert complementarity at score 0.10. Counts sum three paired runs and repeat the same ground-truth (GT) annotations; they are object–seed observations, not independent scenes. Extra recall is expert-only coverage as a percentage of GT, averaged across seeds. Correlations are descriptive seed means. FP: false positive.

Dataset	Both	Primary Only	Expert Only	Neither	Extra Recall	Miss ϕ	FP *r*
Astyx	430	260	59	514	4.67	0.532	0.807
TruckScenes	343	135	116	777	8.46	0.594	0.295
V2X-Radar-V	332	52	37	290	5.20	0.750	0.622
K-Radar	256	25	41	113	9.43	0.664	0.453

**Table 13:** Three-seed progressive analysis. Entries are the mean ± sample SD of ${\mathrm{AP}}_{40}$ across seeds 2026–2028. M1 is candidate-preserving NMS, M2 is residual recovery (RDAR), and M3 is quality-aligned scoring (qflr55). AP: average precision; SD: standard deviation; NMS: non-maximum suppression.

Stage	Astyx	TruckScenes	V2X-Radar-V	K-Radar	Macro
PointPillars	28.5987±1.6869	15.7163±1.7772	40.1987±0.9695	48.9068±1.6606	33.3551±0.2683
+M1	32.8152±1.4692	16.3498±1.7514	41.6866±1.1545	50.5059±2.0589	35.3394±0.3577
+M2	32.8347±1.4689	16.3671±1.7480	41.7029±1.1490	50.5163±2.0546	35.3552±0.3581
+M3	35.8438±1.0680	16.9661±1.2785	44.4122±2.5011	57.6910±2.5304	38.7283±1.2692

**Table 14:** Detection-level calibration in the fixed-expert benchmark (mean ± sample SD over 12 dataset–seed cells). Predictions at score ≥0.10 are greedily matched one-to-one to car ground truth at 3D IoU 0.50. Lower values are better. ECE: expected calibration error; NLL: negative log-likelihood; SD: standard deviation; IoU: intersection-over-union; 3D: three-dimensional.

Method	ECE-15	Adaptive ECE-15	Brier	NLL
Primary	0.1143±0.0067	0.1118±0.0053	0.0672±0.0144	0.2645±0.0350
Oriented WBF	0.1307±0.0399	0.1369±0.0360	0.1581±0.0212	0.4788±0.0490
IoU-score fusion	0.1291±0.0080	0.1274±0.0091	0.0568±0.0061	0.2428±0.0151
Gate	0.1020±0.0147	0.1036±0.0184	0.1012±0.0171	0.3470±0.0409
Gate + voting	0.1029±0.0138	0.1029±0.0167	0.1011±0.0176	0.3470±0.0420

**Table 15:** Leave-one-dataset-out threshold transfer. The held-out dataset is excluded from final grid-point selection. SD is the sample standard deviation across three seeds. AP: average precision.

Held-Out Dataset	$\tau_{v}$	λ	Mean ΔAP ± SD	Minimum	Positive
Astyx	0.22	0.40	+0.5026±0.2998	+0.1931	3/3
TruckScenes	0.22	0.40	+0.5264±0.4764	+0.0252	3/3
V2X-Radar-V	0.24	0.35	+0.2392±0.5728	−0.2945	2/3
K-Radar	0.22	0.40	+1.6836±0.8040	+1.0708	3/3

**Table 16:** Independent seed-holdout threshold transfer. For each dataset, the voting parameters were selected using seeds 2026 and 2027 only, and then evaluated once on the untouched seed 2028. AP: average precision.

Dataset	Selected $\left(\tau_{v},\lambda \right)$	Development Mean ΔAP	Held-Out Seed	Held-Out ΔAP
Astyx	(0.25,0.35)	+1.1287	2028	+0.7313
TruckScenes	(0.25,0.35)	+0.9168	2028	+0.9136
V2X-Radar-V	(0.22,0.40)	+0.1995	2028	+0.8367
K-Radar	(0.24,0.40)	+1.8462	2028	+1.3861

**Table 17:** Selected-proposal geometry changes over three seeds. Counts reuse annotations across seeds and are not independent objects or AP true/false positives. Signed-change counts condition on positive initial overlap; a change tolerance of $10^{-6}$ is used. Lost/gained denote downward/upward crossings of 3D IoU 0.50. AP: average precision; IoU: intersection-over-union; 3D: three-dimensional.

Dataset	Overlapping	Improved	Decreased	Unchanged	Lost	Gained
Astyx	1149	526	621	2	27	34
TruckScenes	1107	590	512	5	31	33
V2X-Radar-V	573	282	291	0	9	9
K-Radar	345	160	180	5	2	3

**Table 18:** Component-level parameter and runtime profile on an NVIDIA GeForce RTX 3090.

**Configuration**	**Parameters**		**Parameter Interpretation**		
RDAR detector	4.830 M		Primary detector and fixed internal baseline		
Quality-aligned detector	4.830 M		M3 changes supervision, not network width		
High-performance detector	4.830 M		Separate trained accuracy-oriented endpoint		
Stable expert detector	4.868 M		Auxiliary detector used by the strict route		
Strict voting operation	+0		Proposal-level operation with no trainable weights		
Profiled stage	Astyx		TruckScenes	V2X-Radar-V	K-Radar
RDAR detector forward	4.740±0.297		3.851±0.311	4.834±1.092	5.887±2.409
High-performance detector forward	8.263±1.163		7.880±1.257	8.124±1.334	8.601±1.471
Strict voting loop	22.7095±0.1031		22.0086±0.1826	22.5393±0.0471	22.8485±0.1735

Hardware: NVIDIA GeForce RTX 3090. Detector-forward profiling uses batch size 4 and excludes AP evaluation, result serialization, expert inference, and prediction-file I/O. Independently profiled rows must not be summed as an end-to-end strict-route latency.

**Table 19:** Loaded-weight detector timing analysis on an NVIDIA GeForce RTX 4060 laptop GPU using K-Radar batch-one inputs. GPU: graphics processing unit; SD: standard deviation; P95: 95th percentile; MiB: mebibytes; M: million parameters.

Component	Parameters	Mean ± SD (ms)	Median	P95	Peak MiB
Primary detector	4.830 M	6.554 ± 1.162	6.182	8.649	150.974
Expert detector	4.868 M	6.591 ± 1.133	6.306	8.386	153.081
Primary + expert sequential	9.698 M	13.106 ± 1.621	12.582	16.326	211.841

Loaded epoch-160 weights; 100 warm-up and 1000 synchronized timed iterations. Rows include native OpenPCDet post-processing; the sequential row executes both models in one process. Detector timings exclude file I/O, gate/vote, and AP evaluation. Vectorized gate/vote matches the original outputs on all 1020 frames (maximum numerical discrepancy $1.44\times 10^{-7}$ ).

## Data Availability

This study analyzes Astyx, MAN TruckScenes, V2X-Radar-V, and K-Radar under the fixed splits and evaluation protocol described in the paper. These datasets are available from their original providers, subject to the respective access and license terms. Derived experimental configurations, result summaries, manuscript sources, and reproducibility materials for the original study are publicly available at the GitHub repository https://github.com/niubisile-wq/sparse-radar-proposal-reliability (accessed on 3 August 2026) and archived at Zenodo (https://doi.org/10.5281/zenodo.21696781). Analysis tables and scripts supporting the reported experiments are included with the reproducibility materials. The original datasets remain subject to provider access and licence terms; raw datasets and model checkpoints are not redistributed.

## References

[1] Paek D.-H., Kong S.-H., Wijaya K.T. (2022). K-Radar: 4D Radar Object Detection for Autonomous Driving in Various Weather Conditions. Adv. Neural Inf. Process. Syst..

[2] Yang L., Zhang X., Li J., Wang C., Song Z., Zhao T., Song Z., Wang L., Zhou M., Shen Y. (2024). V2X-Radar: A Multi-Modal Dataset with 4D Radar for Cooperative Perception. arXiv.

[3] Kong S.-H., Paek D.-H., Lee S. (2025). RTNH+: Enhanced 4D Radar Object Detection Network Using Two-Level Preprocessing and Vertical Encoding. IEEE Trans. Intell. Veh..

[4] Song S.-H., Paek D.-H., Dao M.-Q., Malis E., Kong S.-H. (2026). Enhanced 3-D Object Detection via Diverse Feature Representations of 4-D Radar Tensor. IEEE Sens. J..

[5] Qi C.R., Su H., Mo K., Guibas L.J. PointNet: Deep Learning on Point Sets for 3D Classification and Segmentation. Proceedings of the 2017 IEEE Conference on Computer Vision and Pattern Recognition (CVPR).

[6] Qi C.R., Yi L., Su H., Guibas L.J. (2017). PointNet++: Deep Hierarchical Feature Learning on Point Sets in a Metric Space. Adv. Neural Inf. Process. Syst..

[7] Zhou Y., Tuzel O. VoxelNet: End-to-End Learning for Point Cloud Based 3D Object Detection. Proceedings of the 2018 IEEE/CVF Conference on Computer Vision and Pattern Recognition.

[8] Yan Y., Mao Y., Li B. (2018). SECOND: Sparsely Embedded Convolutional Detection. Sensors.

[9] Lang A.H., Vora S., Caesar H., Zhou L., Yang J., Beijbom O. PointPillars: Fast Encoders for Object Detection from Point Clouds. Proceedings of the 2019 IEEE/CVF Conference on Computer Vision and Pattern Recognition (CVPR).

[10] Wang Y., Jiang Z., Gao X., Hwang J.-N., Xing G., Liu H. RODNet: Radar Object Detection Using Cross-Modal Supervision. Proceedings of the 2021 IEEE Winter Conference on Applications of Computer Vision (WACV).

[11] Nabati R., Qi H. CenterFusion: Center-Based Radar and Camera Fusion for 3D Object Detection. Proceedings of the 2021 IEEE Winter Conference on Applications of Computer Vision (WACV).

[12] Montiel-Marín J., Llamazares A., Antunes M. (2024). Point Cloud Painting for 3D Object Detection with Camera and Automotive 3+1D Radar Fusion. Sensors.

[13] Bodla N., Singh B., Chellappa R., Davis L.S. Soft-NMS—Improving Object Detection with One Line of Code. Proceedings of the 2017 IEEE International Conference on Computer Vision (ICCV).

[14] Li X., Lv C., Wang W., Li G., Yang L., Yang J. (2022). Generalized Focal Loss: Towards Efficient Representation Learning for Dense Object Detection. IEEE Trans. Pattern Anal. Mach. Intell..

[15] Zhang H., Wang Y., Dayoub F., Sünderhauf N. VarifocalNet: An IoU-Aware Dense Object Detector. Proceedings of the 2021 IEEE/CVF Conference on Computer Vision and Pattern Recognition (CVPR).

[16] Patel K., Rambach K., Visentin T., Rusev D., Pfeiffer M., Yang B. Deep Learning-Based Object Classification on Automotive Radar Spectra. Proceedings of the 2019 IEEE Radar Conference (RadarConf).

[17] Graham B., Engelcke M., van der Maaten L. 3D Semantic Segmentation with Submanifold Sparse Convolutional Networks. Proceedings of the 2018 IEEE/CVF Conference on Computer Vision and Pattern Recognition.

[18] Shi S., Wang X., Li H. PointRCNN: 3D Object Proposal Generation and Detection from Point Cloud. Proceedings of the 2019 IEEE/CVF Conference on Computer Vision and Pattern Recognition (CVPR).

[19] Shi S., Guo C., Jiang L., Wang Z., Shi J., Wang X., Li H. PV-RCNN: Point-Voxel Feature Set Abstraction for 3D Object Detection. Proceedings of the 2020 IEEE/CVF Conference on Computer Vision and Pattern Recognition (CVPR).

[20] Yin T., Zhou X., Krähenbühl P. Center-Based 3D Object Detection and Tracking. Proceedings of the 2021 IEEE/CVF Conference on Computer Vision and Pattern Recognition (CVPR).

[21] Musiat A., Reichardt L., Schulze M., Wasenmüller O. (2024). RadarPillars: Efficient Object Detection from 4D Radar Point Clouds. arXiv.

[22] Jia L., Guan R., Zhao H., Zhao Q., Man K.L., Smith J., Yu L., Yue Y. (2025). RadarNeXt: Real-Time and Reliable 3D Object Detector Based On 4D mmWave Imaging Radar. arXiv.

[23] Leitgeb C., Puchleitner T., Ronecker M.P., Watzenig D. (2026). RADE-Net: Robust Attention Network for Radar-Only Object Detection in Adverse Weather. arXiv.

[24] Nuanmeesri S. (2026). Multimodal Fusion Hybrid Attention Parallel Deep Learning for Avocado Ripeness Classification. IEEE Access.

[25] Ren S., He K., Girshick R., Sun J. (2017). Faster R-CNN: Towards Real-Time Object Detection with Region Proposal Networks. IEEE Trans. Pattern Anal. Mach. Intell..

[26] Cai Z., Vasconcelos N. Cascade R-CNN: Delving into High Quality Object Detection. Proceedings of the 2018 IEEE/CVF Conference on Computer Vision and Pattern Recognition.

[27] Qi C.R., Litany O., He K., Guibas L.J. Deep Hough Voting for 3D Object Detection in Point Clouds. Proceedings of the 2019 IEEE/CVF International Conference on Computer Vision (ICCV).

[28] Hosang J., Benenson R., Schiele B. Learning Non-Maximum Suppression. Proceedings of the 2017 IEEE Conference on Computer Vision and Pattern Recognition (CVPR).

[29] Sun P., Zhang R., Jiang Y., Kong T., Xu C., Zhan W., Tomizuka M., Li L., Yuan Z., Wang C. Sparse R-CNN: End-to-End Object Detection with Learnable Proposals. Proceedings of the 2021 IEEE/CVF Conference on Computer Vision and Pattern Recognition (CVPR).

[30] Solovyev R.A., Wang W., Gabruseva T. (2021). Weighted Boxes Fusion: Ensembling Boxes from Different Object Detection Models. Image Vis. Comput..

[31] Tian Z., Shen C., Chen H., He T. FCOS: Fully Convolutional One-Stage Object Detection. Proceedings of the 2019 IEEE/CVF International Conference on Computer Vision (ICCV).

[32] Zhang S., Chi C., Yao Y., Lei Z., Li S.Z. Bridging the Gap Between Anchor-Based and Anchor-Free Detection via Adaptive Training Sample Selection. Proceedings of the 2020 IEEE/CVF Conference on Computer Vision and Pattern Recognition (CVPR).

[33] Lin T.-Y., Goyal P., Girshick R., He K., Dollár P. Focal Loss for Dense Object Detection. Proceedings of the 2017 IEEE International Conference on Computer Vision (ICCV).

[34] Jiang B., Luo R., Mao J., Xiao T., Jiang Y. (2018). Acquisition of Localization Confidence for Accurate Object Detection. European Conference on Computer Vision (ECCV).

[35] Feng C., Zhong Y., Gao Y., Scott M.R., Huang W. TOOD: Task-Aligned One-Stage Object Detection. Proceedings of the 2021 IEEE/CVF International Conference on Computer Vision (ICCV).

[36] Guo C., Pleiss G., Sun Y., Weinberger K.Q. On Calibration of Modern Neural Networks. Proceedings of the 34th International Conference on Machine Learning, PMLR 70.

[37] Küppers F., Kronenberger J., Shantia A., Haselhoff A. Multivariate Confidence Calibration for Object Detection. Proceedings of the 2020 IEEE/CVF Conference on Computer Vision and Pattern Recognition Workshops (CVPRW).

[38] Pathiraja B., Gunawardhana M., Khan M.H. Multiclass Confidence and Localization Calibration for Object Detection. Proceedings of the 2023 IEEE/CVF Conference on Computer Vision and Pattern Recognition (CVPR).

[39] Paek D.-H., Kong S.-H., Wijaya K.T. Enhanced K-Radar: Optimal Density Reduction to Improve Detection Performance and Accessibility of 4D Radar Tensor-Based Object Detection. Proceedings of the IEEE Intelligent Vehicles Symposium (IV).

[40] Palffy A., Pool E., Baratam S., Kooij J.F.P., Gavrila D.M. (2022). Multi-Class Road User Detection with 3+1D Radar in the View-of-Delft Dataset. IEEE Robot. Autom. Lett..

[41] Zheng L., Ma Z., Zhu X., Tan B., Li S., Long K., Sun W., Chen S., Zhang L., Wan M. TJ4DRadSet: A 4D Radar Dataset for Autonomous Driving. Proceedings of the 2022 IEEE 25th International Conference on Intelligent Transportation Systems (ITSC).

[42] Long Y., Kumar A., Morris D., Liu X., Castro M., Chakravarty P. (2023). RADIANT: Radar-Image Association Network for 3D Object Detection. Proc. Aaai Conf. Artif. Intell..

[43] Nobis F., Shafiei E., Karle P., Betz J., Lienkamp M. (2021). Radar Voxel Fusion for 3D Object Detection. Appl. Sci..

[44] Vora S., Lang A.H., Helou B., Beijbom O. PointPainting: Sequential Fusion for 3D Object Detection. Proceedings of the 2020 IEEE/CVF Conference on Computer Vision and Pattern Recognition (CVPR).

[45] Piergiovanni A.J., Casser V., Ryoo M.S., Angelova A. 4D-Net for Learned Multi-Modal Alignment. Proceedings of the 2021 IEEE/CVF International Conference on Computer Vision (ICCV).

[46] Deng J., Chan G., Zhong H., Lu C.X. Robust 3D Object Detection from LiDAR-Radar Point Clouds via Cross-Modal Feature Augmentation. Proceedings of the 2024 IEEE International Conference on Robotics and Automation (ICRA).

[47] Decourt C., VanRullen R., Salle D., Oberlin T. (2024). Leveraging Self-Supervised Instance Contrastive Learning for Radar Object Detection. arXiv.

[48] Meyer M., Kuschk G. (2019). Automotive Radar Dataset for Deep Learning Based 3D Object Detection. 2019 16th European Radar Conference (EuRAD).

[49] Fent F., Kuttenreich F., Ruch F., Rizwin F., Juergens S., Lechermann L., Nissler C., Perl A., Voll U., Yan M. (2024). MAN TruckScenes: A Multimodal Dataset for Autonomous Trucking in Diverse Conditions. Adv. Neural Inf. Process. Syst..

